# Mortality by country of birth in the Nordic countries – a systematic review of the literature

**DOI:** 10.1186/s12889-017-4447-9

**Published:** 2017-05-25

**Authors:** Helena Honkaniemi, Jennie Bacchus-Hertzman, Johan Fritzell, Mikael Rostila

**Affiliations:** 1 Centre for Health Equity Studies, 106 91 Stockholm, Sweden; 2 Aging Research Centre, 113 30 Stockholm, Sweden

**Keywords:** Mortality, Country of birth, Migration, Nordic countries, Sweden, Denmark, Norway, Registry data

## Abstract

**Background:**

Immigration to the Nordic countries has increased in the last decades and foreign-born inhabitants now constitute a considerable part of the region’s population. Several studies suggest poorer self-reported health among foreign-born compared to natives, while results on mortality and life expectancy are inconclusive. To date, few studies have summarized knowledge on mortality differentials by country of birth. This article aims to systematically review previous results on all-cause and cause-specific mortality by country of birth in the Nordic countries.

**Methods:**

The methodology was conducted and documented systematically and transparently using a narrative approach. We identified 43 relevant studies out of 6059 potentially relevant studies in August 2016, 35 of which used Swedish data, 8 Danish and 1 Norwegian.

**Results:**

Our findings from fully-adjusted models on Swedish data support claims of excess mortality risks in specific categories of foreign-born. Most notably, immigrants from other Nordic countries, especially Finland, experience increased risk of mortality from all causes, and specifically by suicide, breast and gynaecological cancers, and circulatory diseases. Increased risks in people from Central and Eastern Europe can also be found. On the contrary, decreased risks for people with Southern European and Middle Eastern origins are found for all-cause, suicide, and breast and gynaecological cancer mortality. The few Danish studies are more difficult to compare, with conflicting results arising in the analysis. Finally, results from the one Norwegian study suggest significantly decreased mortality risks among foreign-born, to be explored in further research.

**Conclusions:**

With new studies being published on mortality differentials between native and foreign-born populations in the Nordic countries, specific risk patterns have begun to arise. Regardless, data from most Nordic countries remains limited, as does the information on specific causes of death. The literature should be expanded in upcoming years to capture associations between country of birth and mortality more clearly.

**Electronic supplementary material:**

The online version of this article (doi:10.1186/s12889-017-4447-9) contains supplementary material, which is available to authorized users.

## Background

Across the world, economic, political and social conflicts have contributed to increased migratory flow, with large groups of people crossing borders in hopes of a better life. According to the United Nations, the number of international immigrants reached an all-time high in 2015 with an estimated 244 million international migrants worldwide [1].

The most commonly reported reasons for immigration to European countries are family- and work-related, with only about 3% immigrating for humanitarian reasons^1^ [2, 3]. However, the proportion of people migrating for various reasons is highly dependent on national migration policies, which differ greatly between European countries. For example, Sweden and Norway have exhibited more open migration policies, with 17% and 11% of immigrants settling there for humanitarian reasons, respectively. In fact, among the OECD countries Sweden receives the greatest portion of humanitarian immigrants [2]. The number of immigrants has increased in all of the Nordic countries since the 1960s, most notably in Sweden [4]. At the beginning of this migratory wave, between 1940 and 1970, the majority of newcomers to Sweden consisted of labour migrants. Since the 1970s, this majority has shifted to refugees and family-related immigrants, although the mid-1990s saw an increase in labour migration once again [5]. Most migrant groups settling in the Nordic countries consist of persons from outside the region, yet the countries also experience a great exchange of people amongst themselves [4].

Much research has been conducted on migration and health in the Nordic countries, with some contradictory results varying by country categorisation and health outcome [6–8]. Examples of earlier review studies include summaries of health among foreign-born populations. In one review of 20 studies, it was found that refugees in Western countries were about ten times more likely to experience post-traumatic stress disorder compared to age-matched general populations in those countries [9]. Another review of 24 studies found that immigrants from countries with particularly high suicide risks, i.e. countries in Northern and Eastern Europe, experienced higher suicide rates relative to groups without a migration background. However, most immigrant groups did not have an increased risk of suicide relative to the native population, with some even experiencing substantially lower risks [10]. Still other studies have employed a broader focus, reviewing literature on all types of health outcomes, without a systematic, transparent and exhaustive approach [6, 7].

Previous findings on mortality differentials by country of birth appear inconclusive. Studies have found increased mortality rates in all-cause mortality and cardiovascular disease within broadly defined groups of foreign-born people compared to the native population [11, 12]. Nevertheless, others have found no significant differences or even lower mortality rates among foreign-born groups in the Nordic countries [8, 13–16]. This sprawling and seemingly convoluted field of migration and health in the Nordic context underpins the need for a comprehensive summary of the field. This is especially warranted given the increasing share of foreign-born individuals in the Nordic countries and the growing number of published articles within the field during the last decades.

This article will focus on the entire foreign-born population in the Nordic countries. All-cause and cause-specific mortality have been chosen as the health outcomes of interest, considering their importance as population health indicators. The work will be guided by the Cochrane and Campbell Collaboration’s principles for systematic reviews [17, 18]. To our knowledge, there have been no earlier systematic reviews summarizing knowledge on mortality differentials between the foreign- and native-born populations in the Nordic countries.

The aim of this article is to systematically collect and summarize knowledge on all-cause and cause-specific mortality differentials between native and foreign-born individuals within the Nordic countries. We hypothesize that mortality differentials between foreign-born and Nordic natives will exist but vary in magnitude and direction, depending on the specific mortality outcome in question and the country or region of birth. Finally, using the reviewed studies as a starting point, we also aim to provide recommendations for future research within the field.

## Methods

This review aims to find and analyze all relevant studies that match the inclusion criteria in a systematic and transparent manner. Handbooks and other literature on meta-analyses and systematic reviews were used as guides [19–21]. Librarian experts were also consulted when developing the search strategy.

### Selection criteria

In this review, peer-reviewed articles that examine mortality differentials (all cause and/or cause-specific mortality) in the Nordic countries between foreign-born and native-born individuals will be included. For the sake of clarity, foreign-born individuals are strictly defined as people who were born in another country and immigrated to a Nordic country, i.e. children of foreign-born were not included in our search criteria. Participants included both women and men, living in any of the Nordic countries, i.e. Denmark, Finland, Iceland, Norway or Sweden, excluding Svalbard, the Faeroe Islands and Greenland. They were either native or foreign-born, working age adults over 18 years old.

Peer-reviewed primary research articles available in the databases^2^ from August 1-15, 2016 were selected. Duplicates were first removed, followed by articles published before 1995 and those written in a language other than English, Swedish or Finnish. Titles and then abstracts were screened using pre-determined selection criteria (see Additional file 1: S2).

During the full-text screening process it was clear that these selection criteria were not sufficient. To maintain quality, only peer-reviewed studies utilizing registry-based data (alone or in conjunction with data from other sources) and with sufficient information in the published study (i.e. table or text with relevant information and the possibility to distinguish relevant results from irrelevant results) were included. Studies where it was not possible to distinguish results between foreign-born and other individuals were excluded, including studies using citizenships, groups where it was not possible to separate foreign-born individuals from their native-born children (“second generation immigrants”), or undefined groups. Foreign-born subjects included immigrants, sometimes categorized with or separately from refugees. Only studies reporting relative mortality risks (as opposed to absolute risks) were included so as to focus on foreign-born and native differentials. Thus, these studies compare mortality risks with a reference category of people with native origins. Finally, included studies either used total population data, data based on representative samples of the total population, or specific subgroups, including patient samples.

### Search strategies

The choice of databases was based on specialist advice and evaluation of the information provider’s coverage^3^ so as to ensure their scope.^4^ The search strings (see Additional file 1: S1) used in the databases are based on an adapted “PICO” search strategy, which is recommended for systematic reviews [19]. The rationale behind it is to identify basic concepts around the population (P), intervention/independent variable (I), comparison (C) and outcome (O).

The following headings were used as the basis for the search string: “Country of birth”, “Mortality” and “Nordic countries”. This approach allowed for thousands of term combinations, resulting in an efficient and comprehensive “net” in the collection process. The next step involved the construction of the search string by finding suitable terms to represent the three headings. A balance between sensitivity (to capture all relevant literature) and accuracy (to avoid irrelevant literature) was the main principle behind the choice of terms [19, 22].

The list of previously known articles was used in this stage by searching for their key words and controlled vocabularies in the information providers.^5^ Each information provider operates in slightly different ways, while the databases have their own technical specialties; for example, MeSH terms are used in PubMed, while ProQuest uses various controlled vocabularies to search different databases. MeSH trees were examined to ensure coverage of all necessary terms without repetition or overlap. Hence, the final search string was modified and tailored to operate in an appropriate and efficient way in each database (see Additional file 1: S1).

Finally, the search string was evaluated using the list of previously known articles in each information provider. The evaluation showed that the search string retrieved all the articles that were accessible in each information provider.

### Collection and screening process

The titles and abstracts of all unique retrieved articles were screened using the selection criteria (see Additional file 1: S2). In the next step, the full texts of potentially relevant articles (i.e. those that either met the inclusion criteria or had too little information to be excluded in this stage) were screened.

### Data and synthesis

Information from the included studies was tabulated into a data extraction form with information primarily on study bibliography, modelling, findings and assessed quality. Quality was ranked as low, medium and high, and was determined based on size and representativeness of study population, detail and rationale behind the foreign countries-of-birth, use of registry-based data, inclusion of control variables, and statistical methods. For synthesizing purposes, subgroupings of the populations and the direction of the results is recommended, so as to organize the results and find patterns among the studies based on the specific mortality outcome [21]. The sizes of the effects were not considered in the analysis, although they have been reported in Additional file 2: Table S1 (see further below). Only the direction was considered – i.e. significant or uncertain (non-significant) increased risk, significant or uncertain (non-significant) decreased risk, or no difference. The results of this compilation were then summarized with a starting point in vote counting technique [19, 23]. The results from the vote counting are further explored and discussed in the narrative analysis and discussion.

Information on results from the respective studies is primarily based on interpretations from the fully-adjusted models of the studies presented in the tables, and secondly based on the authors’ own interpretations of the results.

A systematic review can often result in a meta-analysis, but the current variety and heterogeneity of studies, with regard to populations, outcomes and methodological diversity (such as design and quality), makes the data unsuitable for this technique [19, 20]. The heterogeneity of the selected studies and results also made it difficult to present and compare effect sizes, magnitudes of association, and confidence intervals. Instead, a narrative approach was chosen for the presentation of results [19].

## Results

**Fig. 1.**
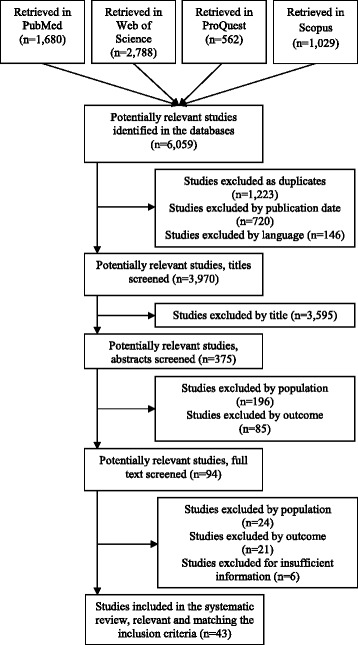
Flow diagram of search process, guided by the PRISMA statement [45]

The selected databases were searched between August 1st and 15th, 2016, generating 6059 retrieved articles in total. The search process for relevant studies is presented in Fig. 1. These were imported to the reference management software EndNote. Since the databases are partly overlapping, 1223 duplicate articles were excluded. An additional 720 and 146 articles were removed based on publication date and language, respectively. The remaining 3970 articles’ titles were screened for relevance, with a substantial proportion being excluded since they were not published in a peer-reviewed journal, did not report primary data, i.e. literature reviews, or were not focused on human populations, i.e. biological, animal or ecological studies. This process was followed by more intensive abstract screening using the selection criteria for the study populations and outcomes, resulting in another 281 excluded studies (Additional file 1: S2).

Full text versions were collected for the remaining 94 studies. However, six of these excluded due to insufficient information on their results, lack of in-text modelling or tables with relevant information. Another 24 studies were excluded because they focused on participants outside the scope of the article, such as undocumented or non-working age migrants, or were based on population groups that were not defined as foreign-born, such as studies examining second generation immigrants, individuals of different language groups and categories based on people’s citizenship. Several of these studies had relevant results that were not possible to distinguish from other populations or countries. Finally, another 21 studies were excluded based on inappropriate or indistinguishable outcome measures. The reasons for exclusion often overlapped, meaning that some studies failed to live up to several inclusion criteria.

The final number of articles that were included and tabulated was 43. 35 of these studies included data on Sweden, eight on Denmark and only one on Norway*.*

### Descriptive information

The descriptive information of the final sample of 43 studies is presented in Additional file 3: Table S2. The table shows that included studies are published between 1995 and 2016, although the full study period, i.e. the time range for data collection and follow-up, ranges from 1958 to 2013 (with one exception going as far back as 1889). Five studies were rated as low quality (note that studies not even deemed low quality were excluded in previous steps), 20 as medium quality and 18 as high quality.

35 of the studies used Swedish data *(S 1-14, 16-23, 27-29, 31-34, 36-38, 40-42),* 6 8 used Danish data *(S 15, 24-26, 30, 31, 35, 39)*, and 1 used Norwegian data *(S 43).* 7 In most of the studies, the aim has been to reach generalizable results for the overall adult population (see Additional file 3: Table S2). However, of the Swedish studies four specifically focused on individuals living in Stockholm *(S 1, 3, 4, 17)* and one in Malmö *(S 20)*. Furthermore, the focus in some of the Swedish studies is on mortality in specific subgroups; i.e. psychiatric inpatients *(S 4)*, survivors of myocardial infarction *(S 17),* people with no history of myocardial infarction *(S 20),* cancer patients *(S 22, 28, 29, 37, 42)* and stroke patients *(S 34).* The results from these studies must then be interpreted as the relative risk of dying as a *patient* and should not be confused with results from the overall populations. Three Danish studies also distinguish between their results for refugees and immigrants *(S 24, 25, 30)*.

Additional file 3: Table S2 shows that all studies included are based on register data, although four also include survey data *(S 5, 8, 9, 13)* and one also includes data from medical reviews *(S 4)*. All studies present results for both women and men, whether combined or separately, with the exception of four studies focusing only on women *(S 19, 23, 27, 29)* and two only on men *(S 22, 28)*.

Sixteen of the studies present results for all-cause mortality *(S 8-10, 13, 14, 17, 18, 21, 22, 25, 27, 36, 37, 39, 41, 43).* Specific causes of death which are reported are circulatory system diseases (including general circulatory disease, ischemic or coronary heart disease, stroke, heart failure and cerebrovascular disease) *(S 8, 18, 20, 25, 27, 31, 33, 34, 39, 41)*, respiratory diseases *(S 39, 41)*, infectious diseases *(S 24, 27, 39)*, accident or unintentional injury *(S 9, 27, 30, 39)*, external causes *(S 27, 36)*, homicide *(S 11, 30, 39)* and others *(S 18, 27, 39, 41)*. Some studies examine multiple specific causes of death. The most studied specific causes of death are suicide *(S 1-7, 9, 12, 15, 16, 27, 30, 38, 39)* and cancer (all-site, site-specific and neoplasms) *(S 18, 19, 22, 23, 25-29, 32, 35, 36, 39-42)*, which are examined in 15 and 16 studies, respectively.

In Additional file 3: Table S2, one can also see that the whole foreign-born population is treated as a uniform category and contrasted to native-born individuals, regardless of their specific country of birth, in 24 of the studies (including the one Norwegian study and one of the Danish studies) *(S 1-7, 9, 15, 17, 19-23, 28-30, 32, 37, 40-43)*. 33 studies report findings for individuals born in specific countries or groups of countries *(S 1, 2, 6, 8-19, 23-36, 38, 39, 42, 43)*.

The categorization of countries and rationale behind the groupings vary among the studies. The most commonly stated motives behind the groupings are geographical origin *(S 8, 12, 13, 16, 17, 23-25, 28, 29, 35, 38, 42),* size of the group from each country of birth *(S 1, 2, 6, 16, 23-25, 31, 32, 35, 36, 43)* and no explicitly stated motivation *(S 3-5, 7, 9, 11, 14, 15, 19-22, 26, 30, 34, 37, 39-41).* There are also examples of rationales such as cultural or economic similarities in the birth countries *(S 6, 8, 16, 27, 31)* and the reliability of available data *(S 10, 18, 31).*

All but four studies involving both sexes *(S 1-4)* use age and sex-matched data (in case-control studies), adjust for these factors in the final model, or stratify the analysis by sex (see Additional file 2: Table S1). In a large proportion of the studies at least one kind of socioeconomic factor is included in the final model, such as general socioeconomic status *(S 12, 16, 17)*, employment/occupation *(S 4, 36)*, education *(S 8, 9, 13, 18, 19, 21, 32, 34, 36-38, 40, 42, 43)*, income *(S 15, 18, 21, 24, 25, 30, 34, 36, 38, 40)*, welfare benefits *(S 12, 18),* housing/residential area *(S 7-9, 12, 34, 38, 40, 42)* and “owning a car” *(S 9)*. Fourteen studies adjust for civil status, marital status or “single parent households” *(S 4, 6-9, 11-13, 15, 16, 18, 38, 40, 43).* A variety of additional factors are also included in other studies such as age, time, geographic origin and health *(S 4, 7, 9, 10, 12, 13, 15-26, 28-43).*

The research designs also vary between studies (see Additional file 3: Table S2). 30 studies use a cohort design *(S 5-10, 12, 13, 16-20, 22, 23, 26-29, 31, 32, 34, 36-43)*, 10 studies use a case-control design *(S 4, 11, 14, 15, 21, 24, 25, 30, 33, 35)* and three studies are based on a cross sectional design *(S 1-3).* The results are presented as Odds Ratios, Hazard Ratios, Standardized Mortality Ratios, Relative Risks, Mortality Rates, Survival/Mortality Rates, Risk/Rate Ratios and results from hypothesis tests (i.e. χ^2^ tests).

### All-cause mortality

Additional file 2: Table S1 show that five studies analyze all-cause mortality risks for all foreign-born individuals collapsed into a separate category and compared to Swedish-born individuals *(S 9, 17, 21, 22, 41)* (also see Additional file 1: S4)*.* Note that results from one study report on all-cause mortality in survivors of myocardial infarction *(S 17)*, in samples specifically excluding patients with a history of myocardial infarction *(S 20)*, and in men with prostate cancer *(S 22)* – these must be interpreted with caution*.* The studies point at an increased mortality risk for the whole foreign-born population *(S 41)*, and more specifically for both sexes *(S 9)*, and only men *(S 17, 21)*. However, in two studies the direction is opposite, indicating a decreased risk in women *(S 17)* and men with prostate cancer *(S 22)*.

When the foreign-born groups are subdivided by country of birth, a more differentiated picture appears *(S 8, 9, 10, 13, 14, 17, 18, 36).* Subgroups are now found in all risk categories, from significantly increased to significantly decreased risks. Swedish results for individuals born in the other Nordic countries *(S 10, 17)*, specifically in Finland *(S 8, 9, 14, 18, 36)* and Denmark *(S 14, 18)*, generally indicate an increased mortality risk. The majority of the results for individuals born in Eastern Europe *(S 8, 10, 17)* and Germany *(S 14, 18, 36)* do not reach significance or do not show a difference. For people from Norway *(S 18, 36)*, Poland *(S 14, 36)*, Former Yugoslavia *(S 14, 18, 36)*, “Western countries” *(S 8, 13)* and Latin America *(S 10, 17)*, the results vary across the board and no clear pattern can be distinguished. Finally, individuals born in Southern Europe primarily exhibit a decreased risk *(S 10, 13).* All of these studies either adjust for or stratify by age and sex.

Other country groupings have not been reported here due to lack of sufficient comparable data (at least two studies per country grouping), but can be seen in Additional file 2: Table S1. This is the case with the two Danish studies examining all-cause mortality *(S 25, 39)*. However, to summarize, the former indicates a decreased risk in immigrants and refugees from most countries (excepting women from Sub-Saharan Africa), while the latter shows an increased mortality risk in all Turkish-born and only male Moroccan-born immigrants.

Only one Norwegian study was found, which examined all-cause mortality *(S 43)*. All foreign-born men and women and the majority of regional and country-specific groupings for men and women indicated significantly decreased all-cause mortality risk. The two exceptions were Nordic women, with no difference in risk; and women from North America or Oceania, with non-significantly decreased risk.

The studies included in this review partly indicate an increased mortality risk among foreign-born people living in Sweden, and specifically among men and some specific groups of foreign-born such as those born in other Nordic countries, including Finland and Denmark. Decreased mortality risks were primarily found in people born in Southern Europe. The results from the two Danish studies are conflicting. Finally, the one Norwegian study presents a significantly decreased risk for immigrants from nearly all country categories.

### Suicide and undetermined death

In Additional file 2: Table S1, nine Swedish studies present results on suicide mortality for the foreign-born population as a whole *(S 1-7, 9, 15)* (also see Additional file 1: S5). In most, the risk of suicide is increased for foreign-born individuals, although this increased risk is only significant in six of them *(S 1-3, 5-7).* Note that results from one study evaluate suicide in a specific group of psychiatric patients *(S 4)*.

Another nine Swedish studies present a more nuanced picture on suicide by specific country or region of birth *(S 1-3, 6, 12, 15, 16, 27, 38)*. For Finnish, Norwegian, Danish, German, Eastern European, Polish, Hungarian and Russian-born individuals, the studies primarily show significant and non-significant increased risks *(S 1, 2, 6, 12, 16)*. No distinguishable patterns can be seen in Western Europeans *(S 6, 12).* Decreased risks are found among individuals born in Southern Europe *(S 6, 12, 16)* and the Middle East *(S 12, 16).* For other countries and country groupings, the results are not comparable between studies, due to unmatched country groupings or the fact that countries are only represented in a single study.

Two out of three studies examining the risk of suicide in Denmark do so for all foreign-born, with contradictory results (one indicating an increased risk, the other suggesting a decreased risk for both refugees and immigrants) *(S 15, 30)*. There is little overlap in the country-specific and regional results *(S 15, 30, 39)*.

To conclude, the reviewed studies suggest increased suicide risks for the foreign-born population in Sweden, as a whole and particularly among individuals born in Northern, Central and Eastern European countries. Meanwhile, lower suicide risks are found among individuals born in Southern Europe and the Middle East. Evidence regarding risks in foreign-born Danes is inconsistent.

### Cancer mortality

A number of the Swedish studies examine cancer mortality differentials, including all-site *(S 18, 32, 41)* and site-specific *(S 19, 22, 23, 28, 29, 32, 40, 42)* cancer, in addition to neoplasm-related *(S 27, 36)* mortality. Note that some studies report risk of cancer mortality specifically within the cancer patient populations *(S 22, 28, 29)*, and thus must be interpreted with caution. This section will present the results for any studies sharing the same cancer diagnosis and categorization of foreign-born.

The risk of all-site cancer mortality is increased for all foreign-born in two Swedish studies, the first for men only and the second for all *(S 32, 41)*. One result indicates no difference in risk for foreign-born women *(S 32)*. Finally, only one study *(S 18)* examines all-site cancer mortality in specific country categories, and thus cannot be analyzed.

Meanwhile, two Swedish studies examining prostate cancer mortality in all foreign-born cancer patients indicate a decreased risk *(S 22, 28).* Only one study examines regional and country-specific results for immigrants, and thus cannot be evaluated here *(S 32)*.

For breast cancer, most evidence shows that all foreign-born experience an increased risk relative to native Swedes *(S 19, 29)*, whether within the total population or specifically as cancer patients, with the exception of one study indicating a decreased risk *(S 32)*. Country- and region-specific results indicate that immigrants from the following areas experience non-significant or significant increased risks of breast cancer mortality: in Northern Europe, specifically Denmark and Iceland; in Central and Western Europe, specifically Former Czechoslovakia, Austria and the Netherlands; and the USA *(S 19, 32)*. Non-significant and significant decreased risks are found in immigrants from Norway; Eastern Europe, in Estonia, Latvia and the Former Soviet Union; Southern Europe as a whole, and specifically Former Yugoslavia, Greece, Italy and Spain; and all of Asia and the Middle East, various sub-regions, including Eastern, Southeastern, Western and South-Central Asia, plus specific countries such as India, Iran, Lebanon and Syria *(S 19, 32)*. All other country- or region-specific results shared between the two studies in question are contradictory.

A handful of studies examine gynaecological cancers, including generally and for specific types. Cervical cancer mortality risks are examined in two Swedish studies *(S 23, 32).* Together, they indicate an increased risk of cervical cancer in all foreign-born compared to the native population. For country-specific results, only immigrants from Norway, Denmark and Southeast Asia indicated increased risks of death from cervical cancer in both studies. A decrease in cervical cancer mortality is found among immigrants born in the UK and Germany, Russia or the Former Soviet Union, Turkey, and Africa as a whole. All other shared results are contradictory. Finally, endometrial and ovarian cancer mortality risks are only examined in one study *(S 23)* and thus could not be compared to any other results.

Due to the specificity of the outcome variables and birth country classifications, the remaining studies on lung *(S 32, 40)*, colon *(S 32)* and stomach *(S 32)* cancers, cutaneous malignant melanoma *(S 42)*, and neoplasms *(S 27, 36)*, cannot be compared and summarized.

In total, four Danish studies examine cancer mortality differentials between foreign-born and native populations *(S 25, 26, 35, 39)*. The only definite result of the vote-counting technique is that risk of all-site cancers is reduced in Turkish-born immigrants *(S 26, 39)*. The remaining studies cannot be evaluated due to incompatible categorizations of the countries of birth or focus on different cancer diagnoses.

The variability in cancer diagnoses and country categorizations (both for the foreign-born and native populations) makes it difficult to effectively compare results on cancer mortality risks across studies. A general pattern of increased breast and gynaecological cancer mortality compared to the Swedish-born population can be seen in immigrants from Northern and Central European countries, although results may be inconsistent depending on the specific country in question and the type of cancer. Decreased risks are commonly found in Southern European and Middle Eastern countries, with the same caveat as above.

### Circulatory system disease mortality

Overall, there are ten studies which examine various circulatory system-related causes of death. Eight of these studies are based on Swedish data *(S 8, 18, 20, 27, 31, 33, 34, 41)* while three are based on Danish data *(S 25, 31, 39)*. Note that one study focuses specifically on a sample of stroke patients *(S 34)*, as opposed to the total population, and thus must be interpreted accordingly.

General circulatory disease mortality is examined in four Swedish studies *(S 8, 27, 31, 36)*. No results are available for all foreign-born. Two of the studies indicate an increased risk of circulatory disease mortality for women and men born in Finland *(S 8, 36)*. Another two report increased risk for Eastern Europeans (women specifically for one, and for all in the other) *(S 8, 31)*. The same studies show increased mortality risks in Eastern Europeans for coronary or ischaemic heart disease as well (once again, for women specifically and for all) *(S 8, 31).* All other birth country categories in the studies are incomparable. Studies reporting cardiovascular disease *(S 18, 41)*, heart failure *(S 20)*, cerebrovascular disease *(S 31)* and stroke *(S 33, 34)* mortality differentials cannot be evaluated here due to lack of comparative data.

Three Danish studies examine circulatory system disease mortality. Among them, they evaluate mortality differentials for general circulatory disease *(S 31)*, cardiovascular disease *(S 25, 39)*, coronary or ischaemic heart disease *(S 31)* and cerebrovascular disease *(S 31)*. Cross-study comparisons were not possible due to lack of data.

To conclude, there are too few studies examining overall circulatory system diseases to derive a pattern in mortality differentials. The only definitive find is that men and women born in Finland and Eastern Europeans in general experience an increased risk in circulatory disease mortality compared to Swedish native populations. In addition, Eastern Europeans experience an increased risk of coronary heart disease mortality. Finally, with three out of eight Danish studies examining these outcomes, there is a clear emphasis on studying circulatory diseases in this context, but comparative data remains limited.

### Other causes of death

Among the remaining causes of death, studies on foreign-born living in Sweden examine homicide, with increased risks for all Non-Nordic and Nordic foreign-born besides Danish, Icelandic and Norwegian males *(S 11)*. Accidents or unintentional injuries and external causes of death are seen in a few studies, with conflicting results *(S 27, 36).* One study examines multiple causes of death, including infectious or parasitic diseases, HIV, mental and behavioural diseases, and perinatal diseases, and examines foreign-born as categorized by a low-, medium- or high-income birth country – across the board, most experience increased mortality risks in these categories *(S 27)*. Another study reports significantly increased risk of mortality from diabetes and chronic respiratory diseases for all foreign-born living in Sweden *(S 41)*. “Other causes” of mortality *(S 18, 27, 41)* cannot be summarized due to inconsistent categorization of causes.

Among the remaining Danish studies, one examines infectious disease mortality for both refugees and immigrants, with increased risk of death for all foreign-born categories other than Middle Eastern and Iraqi refugee men and Asian immigrant females, who experience non-significant decreased risks *(S 24)*. Another study reporting on infectious disease mortality risks has more inconclusive evidence *(S 39)*. Two Danish studies examine the risk of homicide, with one study presenting an increased risk of death in refugees but a more unclear pattern among immigrants *(S 30)* and the other reporting increased risks specifically for Turkish- and Moroccan-born immigrants *(S 39)*. Both also compare mortality risks for unintentional injuries, with significantly decreased risks for all foreign-born and specifically Turkish- and Moroccan-born immigrants *(S 30, 39)*. Finally, respiratory disease mortality is examined in only one study *(S 39)*, with non-significantly decreased mortality risks for Turkish- and Moroccan-born.

Overall, it is difficult to summarize evidence on mortality from specific causes since results depend on cause of death and migrant group studied. Based on the reviewed studies there is no clear pattern of increased cause-specific mortality in the total foreign-born population in Sweden or Denmark, although increased cause-specific mortality risks for certain foreign-born groups have been found.

## Discussion

The aim of this review was to systematically collect and summarize knowledge on mortality differentials between native and foreign-born individuals within the Nordic countries, and to explore certain qualities of earlier research that were important for the understanding of mortality inequalities. Our search identified 43 relevant studies.

In Sweden, we found an increased risk of all-cause mortality in the foreign-born population as a whole, and specifically in some foreign-born groups such as those born in the Nordic countries, including Finland and Denmark. On the contrary, immigrants from Southern Europe exhibited decreased risks. Similar results arose for suicide mortality, with increased risks for all foreign-born and people born in Northern, Central and Eastern Europe, and decreased risks for Southern European and Middle Eastern immigrants. Limited evidence suggests an increased risk of all-site cancer mortality but decreased risk for prostate cancer in all foreign-born. Increased risk of breast cancer mortality was found among people from other Northern European countries, Central and Western European countries and the USA, while decreased risks were found in Eastern and Southern Europe in addition to the Middle East and Asia. Gynaecological cancer risks (specifically cervical) were exclusively increased for Danish and Norwegian immigrants. Finally, circulatory disease mortality risk was found to be increased for Finnish immigrants. The results clearly showed the importance of studying more specific groups of foreign-born rather than lumping countries into broad categories, since mortality risks varied greatly by country of birth. As for Danish studies, the results were much more conflicting. Evidence from the one Norwegian study indicated a decreased mortality risk for most foreign-born categories.

The various research designs in our reviewed studies made it difficult to pinpoint specific birth countries with similar mortality risks for all-cause or cause-specific mortality. In general, most Swedish studies reported on immigrants from other Nordic countries, with 17 studies on Finnish immigrants *(S 1, 2, 6, 8, 9, 11-14, 16-19, 23, 32, 33, 36)*, 10 Danish and Norwegian *(S 1, 2, 6, 11, 14, 18, 19, 23, 32, 36)* and six Nordic in general *(S 10, 19, 32, 34, 38, 42)*. Many of the Danish studies focused instead on Middle Eastern populations *(S 24, 25, 30, 31, 35)*, and specifically Turkish- *(S 26, 39)* and Moroccan-born *(S 39)* immigrant groups. It is possible that these categories have been selected based on traditional groupings which have persisted into current research efforts. Thus, other categories have not been examined enough in the past to ascertain their differences. With this limited evidence, other cross-country comparisons were less feasible.

Overall, the quality of the studies has increased with time. The few cross-sectional studies were conducted early on in the 1990s. Since then, cohort studies with various control variables and case-control studies have become more common, most likely due to increased access to registry data. With this sample sizes have grown, often to cover the entire population. A third pattern can be seen in the increased use of Cox regression or proportional hazards models over logistic regressions or other simpler statistical methods, the former being a superior method to examining mortality events. Finally, many of the earlier studies focused specifically on suicide risks, while the more recent studies have begun to examine all-cause and multiple cause-specific mortality differentials instead. Despite the improved quality, there is still great variability in the design of these studies, and thus a meta-analysis is not yet recommendable.

Overall, although increased mortality risks in some specific groups of foreign-born were found, the findings from this systematic review provide conflicting evidence on all-cause and cause-specific mortality among the general foreign-born population. This stresses the importance of avoiding broad birth country categories, an issue that has been pointed out in recent research [8]. It also raises the question about potential explanations for relatively advantaged health situations among specific groups of migrants.

Two main theories for lower mortality risks in some groups of foreign-born have been proposed. The first, the “salmon bias hypothesis”, refers to statistical and technical data issues arising from the tendency for old and ill people to return to their birth countries before they die [24]. These deaths are not registered in the country they leave, rendering the immigrants “statistically immortal”. If not reported to the authorities, this can create bias in studies on migration and mortality [25]. One example of this effect can be found in a study by Weitoft et al. [26]. When controlling for income during a follow-up period of mortality in groups of immigrants, the researchers found that negative coefficients that were previously significant for some groups were no longer significant, indicating that many individuals may have left the country without this being registered [26]. Next, the “healthy migrant hypothesis” explains the health advantages of foreign-born groups via certain selection processes. Simply put, the health and socioeconomic advantages of certain groups create more opportunities for migration, thus contributing to lower mortality and better health among said groups in the new country, compared to the population in their birth country [24].

A large portion of the literature suggests that the variation in mortality risks among foreign-born groups is also shaped by gender [8]. One report in particular emphasizes the need for gender perspectives in health research on foreign-born, highlighting the vulnerability women experience in recipient countries and their increased risk of being unemployed, socially isolated and exposed to discrimination [27]. However, protective factors have also been suggested among some groups of foreign-born women, such as lower alcohol and tobacco use [15, 28]. The evidence for sex differences in this review is conflicting, stressing the need for further research.

To add to this, it would be interesting to examine mortality differentials in undocumented migrants and see if their immigration status reflects a poorer socioeconomic and health status, as has already been done in at least one study [29]. Studies comparing refugee and immigrant health outcomes would also shed light on some of the reasoning behind existing mortality differentials. Another important task for future research is to investigate whether inequalities in health and mortality continue to exist among children of foreign-born individuals. In fact, some findings from the US have suggested that mortality risks increase in these children [24, 25], while evidence for changed suicide and gynaecological cancer mortality risks in second-generation immigrants in Sweden also exists [30, 31]. Based on the findings from this review there is also a clear need to stimulate more research within the field in the Nordic countries besides Sweden, and to compare mortality risks by country of birth between the Nordic countries using a comparative approach. Such studies could provide important context-specific information regarding differences in conditions and policies between the Nordic countries that could potentially buffer adverse health among foreign-born individuals.

Other key factors to consider in future studies include mechanisms linking country of birth and mortality, such as those related to genetic or biological differences, the country of origin and act of migration, and conditions in the new country. Most of the studies included in this review have empirically adjusted for the “standard” confounders or mediators (for example, employment or occupation, education, income, housing, marital status, etc.) while few studies have specifically raised interest in causal pathways linking migration and health. A variety of explanations for excess mortality among immigrants emphasize biological and genetic differences concerning susceptibility and vulnerability to disease and ill health [32]. Others suggest that previous experiences in the country of birth (i.e. war and conflict), exposure to infectious diseases and stressors associated with the act of migration might influence the health of immigrants [33]. Still others claim that conditions in the new country such as scarcity of social capital, poor integration and discrimination might account for the reduced health of migrants [34–36]. The contribution of social conditions has received particular interest in earlier research, as their link with health is well established, and generally foreign-born populations experience worse conditions than native-born ones [8, 37]. For example, Fritzell et al. [38] showed that poverty was much higher among immigrants in the Nordic countries, and among those born outside the EU, poverty rates were more than double that of the overall population.

Finally, it is important to consider that the composition of the migrant population is constantly changing in the Nordic countries due to new conflicts, wars, economic crises, and natural disasters, etc. in the world – with this, more studies in the Nordics outside of Sweden are now being published. Continuously updated information on health and mortality among these newly arrived groups is hence strongly suggested.

### Strengths and weaknesses

Some methodological challenges for studies within the field of immigration and health have previously been discussed [39]. The primary concern is the “oversimplification” of foreign-born populations, that is, the handling of significantly heterogeneous groups as homogenous via poorly defined ethnic categorizations. This review highlights this problem and shows that it may lead to the masking of potential health differentials.

The simple presentation of the results inspired by the vote-counting technique is a limitation of this review. However, with the reporting of effect sizes, magnitudes of association, confidence intervals, quality and general context of the reviewed studies, readers can individually interpret the results [23, 40]. Our aim to simply study the existence of increased or decreased risks of all-cause or cause-specific mortality was motivated by the fact that the variety of results was otherwise difficult to summarize and input into a meta-analysis. Despite this, the Cochrane Collaboration suggests systematic reviews to be updated every 2 years, to achieve the high quality provision of current evidence [20]. Complementary searches should thus be conducted in the future to capture new evidence in this growing field, which could make meta-analyses more feasible.

It is strongly recommended that at least two persons screen the articles and extract all the data independently, comparing their findings afterwards, so as to reduce errors and potential biases. Furthermore, there are special recommendations for dealing with potential disagreements [20]. This might have increased the likelihood of random errors and systematic bias. Nevertheless, each step in the search and screening process was discussed regularly with the rest of the co-authors in order to minimize this risk.

One strength of this review is the fact that all studies are peer-reviewed and primarily based on register data, potentially limiting issues of selection bias. However, some earlier studies indicate a higher risk of misclassified causes of death among foreign-born individuals compared to native-born individuals [41]. This would only be of major concern if the aim was to focus on specific diagnostic causes of death, but of little consequence when focusing on all-cause mortality. Another potential problem of this review is that some of the included studies were based on patient groups or clinical samples rather than total population data. The results from these studies should then be interpreted as the relative risk of dying as a patient and thus not be confused with results from studies based on total populations. However, one might argue that the total population studies are also difficult to compare due to different designs, included variables, etc. Finally, different definitions of “immigrants” were used in the studies, with some including refugees and others reporting data on both separately – these must also be interpreted carefully to avoid making assumptions about the wrong foreign-born groups.

Another source of bias could be associated with “over-coverage”, where foreign-born individuals return to their country of origin without this being registered by authorities in total-population registries [26]. Assuming that a considerable number of individuals leave the receiving country without this being registered, there is a chance that the mortality risk among foreign-born individuals will be underestimated. Consequently, we may have underestimated the resulting health inequalities by country of birth within this review.

The results from previous studies may be difficult to compare since the potential confounders they account for vary extensively. The interpreted results in Additional file 2: Table S1 were based on the fully-adjusted final models, but these models account for different controls – thus, depending on the control variables used, the final results may also vary. Furthermore, it may be difficult to compare empirical findings across time, since the composition of the regional foreign-born populations in the Nordic countries is constantly changing as new groups of immigrants arrive – for instance, groups of Middle Eastern migrants may be more composed of people from one specific country than they have in the past. Similarly, researchers must account for the changing composition of immigrant populations relating to different reasons for leaving their countries, whether for labour, family or humanitarian reasons.

In addition, there is a greater tendency for significant and positive results to be published than non-significant results [42]. This would imply that potential studies with non-significant results regarding mortality risk differentials would be underestimated, for both the whole foreign-born population and for individuals born in specific countries. It is therefore recommendable to search for unpublished works as well so as to minimize the impact of this issue, and to detect any potential differences with a funnel plot.

Finally, the large number of Swedish studies included in this review and the much lower number of studies from other Nordic countries deserves some discussion. Reasons for the lack of non-Swedish studies could be related to the inclusion criteria (see Additional file 1: S2). One restriction to only include peer-reviewed studies would have excluded so-called grey literature, such as governmental and agency reports, working papers and the like. It is possible that the majority of studies in other Nordic countries are not peer-reviewed and therefore not included within this review. However, the databases searched in this review could have only returned a limited scope of these reports, thus biasing our results. Furthermore, our choice to conduct the search in English, Swedish and Finnish would have more likely biased the findings within the grey literature, since reports are more likely to be written in the country’s native language, compared to international, peer-reviewed articles.

The absence of multiple Norwegian studies in the final collection should not be interpreted as a lack of knowledge on health among foreign-born individuals in Norway. Attanapola, for instance, indicated that a substantial number of studies have been published concerning health outcomes such as mental health, diet and lifestyle-related health problems, reproductive health problems and infectious diseases [6]. However, none of these addressed mortality as a health outcome. Since the one included Norwegian article is also the most recently published article in our review, it is possible that more studies on this subject will follow soon.

As for the complete lack of Finnish studies, these are partly explained by one inclusion criteria – comparison of populations by country of birth. The majority of Finnish literature focuses on mortality differentials and other health outcomes across regions, and more significantly by language – that is, between the Finnish- and Swedish-speaking populations [43]. Another focus is twin studies, where health outcomes have been compared between separated twins living in Finland and Sweden, for one example [44]. Thus, evidence on people born outside of Finland is lacking. A third possible explanation, which also relates to the lack of studies on Iceland, is simply the lack of sufficiently large immigrant populations in both countries. Despite recent increases in immigration to Finland and Iceland, the nature of mortality studies means that not enough time has passed yet to capture longitudinal differences between foreign- and native-born mortality differentials.

## Conclusions

The results support the existence of inequalities in mortality risk between native and foreign-born individuals. In general, increased all-cause and certain cause-specific mortality risks in Sweden are found among immigrants born in Northern, Central and Eastern Europe, most notably in Finland, while decreased risks are found for immigrants from Southern Europe and the Middle East. Much of the evidence for the very specific causes or birth countries was conflicting or limited, especially in the Danish studies. Overall, results for specific countries and regions contribute to a collective picture of significantly heterogeneous mortality risks among foreign-born individuals, compared to native-born populations.

Despite the large number of studies included in this study, the variability in inspected birth countries, native Nordic countries and specific causes of death means that only a small fragment of the studies are comparable. This review can be utilized as a preliminary source of evidence for mortality differentials, and as a guide for other researchers to identify knowledge gaps in the literature and inform further studies.

## Additional files

Additional file 1: **S1.** Search Strings. **S2.** Selection Criteria. **S3.** Reference list of included articles. **S4.** Results from studies analysing all-cause mortality in Sweden, for partly overlapping gender and country subgroups. **S5.** Results from studies analysing suicide and undetermined death in Sweden, for partly overlapping gender and country subgroups. (DOCX 49 kb)

Additional file 2: Table S1. Summary of descriptive information for included studies (See Additional file 1: S3 for a reference list of studies included). (DOCX 50 kb)

Additional file 3: Table S2. Summary of models and results for included studies (See Additional file 1: S3 for a reference list of studies included). (DOCX 102 kb)

## Footnotes

1 “Persons who have completed the asylum procedure with a positive outcome and have been granted some sort of protection (refugee status or another form of protection) or have been resettled through programmes outside the asylum procedure.”

2 Databases include: PubMed (Medline); databases from the interface Web Of Science (Science Citation Index Expanded (SCI-EXPANDED) (1945-Present), Social Sciences Citation Index (SSCI) (1956-Present), Arts & Humanities Citation Index (A&HCI) (1975-Present); databases from the interface ProQuest LLC (Education Resources Information Center (ERIC), International Bibliography of Social Sciences (IBSS), Published International Literature on Traumatic Stress (PILOTS), PsycARTICLES, PsycINFO, Social Services Abstracts, Sociological Abstracts); and Scopus.

3 The evaluation was built on a list of 17 previously known articles that fulfil the review’s inclusion criteria. This list has been checked against the retrieved articles in the databases to ensure that the search strategy generates the type of articles that we were searching for. The previously known articles were: 1-17, seen in “Reference list of included articles” in Additional file 1: S3.

4 The evaluation shows that PubMed (including Medline), Web Of Science (including: Science Citation Index Expanded, Social Sciences Citation Index, Arts & Humanities Citation Index), ProQuest LLC (including: Education Resources Information Center, International Bibliography of Social Sciences, Published International Literature on Traumatic Stress, PsycARTICLES, PsycINFO, Social Services Abstracts, Sociological Abstracts), and Scopus cover 100% of the list of previously known articles and were therefore chosen.

5 MeSH (Medical Subject Headings) in PubMed.

6 Note: All numbers that are italicized, preceded by an S, and enclosed in the same parentheses refer to the reviewed studies, listed in the Additional file 1.

7 Note: One study (*S 39)* reports data from both Sweden and Denmark.
