# Supplementary material for: Mortality by country of birth in the Nordic countries – a systematic review of the literature
Source: BMC Public Health. 2017 May 25;17:511. doi: 10.1186/s12889-017-4447-9 (PMC5445314; doi:10.1186/s12889-017-4447-9)
Supplement: Supplementary file 1 — S1. Search Strings. S2. Selection Criteria. S3. Reference list of included articles. S4. Results from studies analysing all-cause mortality in Sweden, for partly overlapping gender and country subgroups. S5. Results from studies analysing suicide and undetermined death in Sweden, for partly overlapping gender and country subgroups. (DOCX 49 kb) [file 12889_2017_4447_MOESM1_ESM.docx]

## Additional file 1

## S1. Search Strings

**Search string template:**

Country of birth AND Mortality AND Nordic countries (All terms representing these concepts are combined with “OR”)

##

## Search string in Pub Med:

Search ((((((asylum* AND (Humans[Mesh])) OR ("country of birth" AND (Humans[Mesh])) OR ("country of origin" AND (Humans[Mesh])) OR ("countries of origin" AND (Humans[Mesh])) OR (emigra* AND (Humans[Mesh])) OR (ethnic* AND (Humans[Mesh])) OR (expat* AND (Humans[Mesh])) OR ("foreign background" AND (Humans[Mesh])) OR ("foreign backgrounds" AND (Humans[Mesh])) OR ("foreign born" AND (Humans[Mesh])) OR (foreigner* AND (Humans[Mesh])) OR (immigra* AND (Humans[Mesh])) OR (migrant* AND (Humans[Mesh])) OR ("minority group" AND (Humans[Mesh])) OR ("minority groups" AND (Humans[Mesh])) OR ("language group" AND (Humans[Mesh])) OR ("language groups" AND (Humans[Mesh])) OR (race* AND (Humans[Mesh])) OR (racial* AND (Humans[Mesh])) OR (refugee* AND (Humans[Mesh]))) AND (Humans[Mesh])) OR ((("Emigrants and Immigrants"[Mesh] AND (Humans[Mesh])) OR ("Emigration and Immigration"[Mesh] AND (Humans[Mesh])) OR ("Minority Groups"[Mesh] AND (Humans[Mesh])) OR ("Minority Health"[Mesh] AND (Humans[Mesh])) OR ("Population Groups"[Mesh] AND (Humans[Mesh])) OR ("Race Relations"[Mesh] AND (Humans[Mesh])) OR ("Refugees"[Mesh] AND (Humans[Mesh])) OR ("Transients and Migrants"[Mesh] AND (Humans[Mesh]))) AND (Humans[Mesh]))) AND (Humans[Mesh])) AND (((((alive* AND (Humans[Mesh])) OR (corpse* AND (Humans[Mesh])) OR (dead* AND (Humans[Mesh])) OR (death* AND (Humans[Mesh])) OR (deceas* AND (Humans[Mesh])) OR (decedent* AND (Humans[Mesh])) OR (dying* AND (Humans[Mesh])) OR (fatal* AND (Humans[Mesh])) OR (homicid* AND (Humans[Mesh])) OR (kill* AND (Humans[Mesh])) OR (lethal* AND (Humans[Mesh])) OR ("life expectancy" AND (Humans[Mesh])) OR (longevit* AND (Humans[Mesh])) OR (manslaughter* AND (Humans[Mesh])) OR (mortal* AND (Humans[Mesh])) OR (murder* AND (Humans[Mesh])) OR (suicid* AND (Humans[Mesh])) OR (surviv* AND (Humans[Mesh]))) AND (Humans[Mesh])) OR ((("Mortality"[Mesh] AND (Humans[Mesh])) OR ("Life Expectancy"[Mesh] AND (Humans[Mesh])) OR ("Life Tables"[Mesh] AND (Humans[Mesh])) OR ("Longevity"[Mesh] AND (Humans[Mesh])) OR ("Death Certificates"[Mesh] AND (Humans[Mesh])) OR ("Homicide"[Mesh] AND (Humans[Mesh])) OR ("Death"[Mesh] AND (Humans[Mesh])) OR ("Suicide"[Mesh] AND (Humans[Mesh])) OR ("Survival"[Mesh] AND (Humans[Mesh]))) AND (Humans[Mesh]))) AND (Humans[Mesh])) AND (((((dane* AND (Humans[Mesh])) OR (danish* AND (Humans[Mesh])) OR (denmark* AND (Humans[Mesh])) OR (finland* AND (Humans[Mesh])) OR (finnish* AND (Humans[Mesh])) OR (finn* AND (Humans[Mesh])) OR (iceland* AND (Humans[Mesh])) OR (nordic* AND (Humans[Mesh])) OR (norw* AND (Humans[Mesh])) OR (scandinav* AND (Humans[Mesh])) OR (swed* AND (Humans[Mesh]))) AND (Humans[Mesh])) OR (("Scandinavian and Nordic Countries"[Mesh] AND (Humans[Mesh])))AND (Humans[Mesh])) AND (Humans[Mesh]))) Limits: Humans

Search string in Web of Science:

**Sökning Antal träffar Söksträng**

# 51 2,788 #50 AND #38 AND #18

Databases=SCI-EXPANDED, SSCI, A&HCI Timespan=All Years

# 50 317,871 #49 OR #48 OR #47 OR #46 OR #45 OR #44 OR #43 OR #42 OR #41 OR # 40 OR #39

Databases=SCI-EXPANDED, SSCI, A&HCI Timespan=All Years

# 49 106,090 Topic=(swed*)

Databases=SCI-EXPANDED, SSCI, A&HCI Timespan=All Years

# 48 16,930 Topic=(scandinav*)

Databases=SCI-EXPANDED, SSCI, A&HCI Timespan=All Years

# 47 83,893 Topic=(norw*)

Databases=SCI-EXPANDED, SSCI, A&HCI Timespan=All Years

# 46 11,336 Topic=(nordic*)

Databases=SCI-EXPANDED, SSCI, A&HCI Timespan=All Years

# 45 15,850 Topic=(iceland*)

Databases=SCI-EXPANDED, SSCI, A&HCI Timespan=All Years

# 44 2,493 Topic=(finns*)

Databases=SCI-EXPANDED, SSCI, A&HCI Timespan=All Years

# 43 26,425 Topic=(finnish*)

Databases=SCI-EXPANDED, SSCI, A&HCI Timespan=All Years

# 42 40,704 Topic=(finland*)

Databases=SCI-EXPANDED, SSCI, A&HCI Timespan=All Years

# 41 30,817 Topic=(denmark*)

Databases=SCI-EXPANDED, SSCI, A&HCI Timespan=All Years

# 40 31,404 Topic=(danish*)

Databases=SCI-EXPANDED, SSCI, A&HCI Timespan=All Years

# 39 3,375 Topic=(dane*)

Databases=SCI-EXPANDED, SSCI, A&HCI Timespan=All Years

# 38 3,592,614 #37 OR #36 OR #35 OR #34 OR #33 OR #32 OR #31 OR #30 OR #29

OR #28 OR #27 OR #26 OR #25 OR #24 OR #23 OR #22 OR #21 OR #20 OR #19

Databases=SCI-EXPANDED, SSCI, A&HCI Timespan=All Years

# 37 1,022,414 Topic=(surviv*)

Databases=SCI-EXPANDED, SSCI, A&HCI Timespan=All Years

# 36 58,476 Topic=(suicide*)

Databases=SCI-EXPANDED, SSCI, A&HCI Timespan=All Years

# 35 12,334 Topic=(murder*)

Databases=SCI-EXPANDED, SSCI, A&HCI Timespan=All Years

# 34 659,571 Topic=(mortal*)

Databases=SCI-EXPANDED, SSCI, A&HCI Timespan=All Years

# 33 465 Topic=(manslaughter*)

Databases=SCI-EXPANDED, SSCI, A&HCI Timespan=All Years

# 32 40,334 Topic=(longevit*)

Databases=SCI-EXPANDED, SSCI, A&HCI Timespan=All Years

# 31 21,478 Topic=("life expectanc*")

Databases=SCI-EXPANDED, SSCI, A&HCI Timespan=All Years

# 30 108,251 Topic=(lethal*)

Databases=SCI-EXPANDED, SSCI, A&HCI Timespan=All Years

# 29 193,922 Topic=(kill*)

Databases=SCI-EXPANDED, SSCI, A&HCI Timespan=All Years

# 28 9,631 Topic=(homicid*)

Databases=SCI-EXPANDED, SSCI, A&HCI Timespan=All Years

# 27 101,276 Topic=(fatal*)

Databases=SCI-EXPANDED, SSCI, A&HCI Timespan=All Years

# 26 27,390 Topic=(dying*)

Databases=SCI-EXPANDED, SSCI, A&HCI Timespan=All Years

# 25 1,287,889 Topic=(die*)

Databases=SCI-EXPANDED, SSCI, A&HCI Timespan=All Years

# 24 1,873 Topic=(decedent*)

Databases=SCI-EXPANDED, SSCI, A&HCI Timespan=All Years

# 23 14,372 Topic=(deceas*)

Databases=SCI-EXPANDED, SSCI, A&HCI Timespan=All Years

# 22 680,264 Topic=(death*)

Databases=SCI-EXPANDED, SSCI, A&HCI Timespan=All Years

# 21 101,273 Topic=(dead*)

Databases=SCI-EXPANDED, SSCI, A&HCI Timespan=All Years

# 20 2,991 Topic=(corpse*)

Databases=SCI-EXPANDED, SSCI, A&HCI Timespan=All Years

# 19 37,578 Topic=(alive*)

Databases=SCI-EXPANDED, SSCI, A&HCI Timespan=All Years

# 18 835,983 #17 OR #16 OR #15 OR #14 OR #13 OR #12 OR #11 OR #10 OR #9

OR #8 OR #7 OR #6 OR #5 OR #4 OR #3 OR #2 OR #1

Databases=SCI-EXPANDED, SSCI, A&HCI Timespan=All Years

# 17 14,804 Topic=(refugee*)

Databases=SCI-EXPANDED, SSCI, A&HCI Timespan=All Years

# 16 62,662 Topic=(racial*)

Databases=SCI-EXPANDED, SSCI, A&HCI Timespan=All Years

# 15 194,914 Topic=(race*)

Databases=SCI-EXPANDED, SSCI, A&HCI Timespan=All Years

# 14 803 Topic=("language group*")

Databases=SCI-EXPANDED, SSCI, A&HCI Timespan=All Years

# 13 6,310 Topic=("minority group*")

Databases=SCI-EXPANDED, SSCI, A&HCI Timespan=All Years

# 12 406,944 Topic=(migrat*)

Databases=SCI-EXPANDED, SSCI, A&HCI Timespan=All Years

# 11 31,912 Topic=(migrant*)

Databases=SCI-EXPANDED, SSCI, A&HCI Timespan=All Years

# 10 63,949 Topic=(immigra*)

Databases=SCI-EXPANDED, SSCI, A&HCI Timespan=All Years

# 9 3,565 Topic=(foreigner*)

Databases=SCI-EXPANDED, SSCI, A&HCI Timespan=All Years

# 8 3,376 Topic=("foreign born*")

Databases=SCI-EXPANDED, SSCI, A&HCI Timespan=All Years

# 7 52 Topic=("foreign background*")

Databases=SCI-EXPANDED, SSCI, A&HCI Timespan=All Years

# 6 3,225 Topic=(expat*)

Databases=SCI-EXPANDED, SSCI, A&HCI Timespan=All Years

# 5 153,451 Topic=(ethnic*)

Databases=SCI-EXPANDED, SSCI, A&HCI Timespan=All Years

# 4 14,243 Topic=(emigra*)

Databases=SCI-EXPANDED, SSCI, A&HCI Timespan=All Years

# 3 4,993 Topic=("countr* of origin*")

Databases=SCI-EXPANDED, SSCI, A&HCI Timespan=All Years

# 2 1,351 Topic=("countr* of birth*")

Databases=SCI-EXPANDED, SSCI, A&HCI Timespan=All Years

# 1 5,333 Topic=(asylum*)

Databases=SCI-EXPANDED, SSCI, A&HCI Timespan=All Years

Search string in ProQuest LLC:

TI((asylum* OR "countr* of birth*" OR "countr* of origin*" OR emigr* OR ethnic* OR expat* OR "foreign background*" OR "foreign born*" OR foreigner* OR immigra* OR migrant* OR migrat* OR "minority group*" OR "language group*" OR race* OR racial* OR refugee*) AND (alive* OR corpse* OR die* OR dead* OR death* OR deceas* OR decedent* OR dying* OR fatal* OR homicid* OR kill* OR lethal* OR "life expectanc*" OR longevit* OR manslaughter* OR mortal* OR murder* OR suicid* OR suviv*) AND (dane* OR danish* OR denmark* OR finland* OR finn* OR iceland* OR nordic* OR norw* OR scandinavia* OR swed*)) OR AB((asylum* OR "countr* of birth*" OR "countr* of origin*" OR emigr* OR ethnic* OR expat* OR "foreign background*" OR "foreign born*" OR foreigner* OR immigra* OR migrant* OR migrat* OR "minority group*" OR "language group*" OR race* OR racial* OR refugee*) AND (alive* OR corpse* OR die* OR dead* OR death* OR deceas* OR decedent* OR dying* OR fatal* OR homicid* OR kill* OR lethal* OR "life expectanc*" OR longevit* OR manslaughter* OR mortal* OR murder* OR suicid* OR suviv*) AND (dane* OR danish* OR denmark* OR finland* OR finn* OR iceland* OR nordic* OR norw* OR scandinavia* OR swed*)) OR SU((asylum* OR "countr* of birth*" OR "countr* of origin*" OR emigr* OR ethnic* OR expat* OR "foreign background*" OR "foreign born*" OR foreigner* OR immigra* OR migrant* OR migrat* OR "minority group*" OR "language group*" OR race* OR racial* OR refugee*) AND (alive* OR corpse* OR die* OR dead* OR death* OR deceas* OR decedent* OR dying* OR fatal* OR homicid* OR kill* OR lethal* OR "life expectanc*" OR longevit* OR manslaughter* OR mortal* OR murder* OR suicid* OR suviv*) AND (dane* OR danish* OR denmark* OR finland* OR finn* OR iceland* OR nordic* OR norw* OR scandinavia* OR swed*))

**Search string in Scopus:**

INDEXTERMS ( asylum* OR "countr* of birth*" OR "countr* of origin*" OR emigr* OR ethnic* OR expat* OR "foreign background*" OR "foreign born*" OR foreigner* OR immigra* OR migrant* OR migrat* OR "minority group*" OR "language group*" OR race* OR racial* OR refugee* ) AND INDEXTERMS ( alive* OR corpse* OR die* OR dead* OR death* OR deceas* OR decedent* OR dying* OR fatal* OR homicid* OR kill* OR lethal* OR "life expectanc*" OR longevit* OR manslaughter* OR mortal* OR murder* OR suicid* OR suviv* ) AND INDEXTERMS ( dane* OR danish* OR denmark* OR finland* OR finn* OR iceland* OR nordic* OR norw* OR scandinavia* OR swed* )

| S2. Selection Criteria | | | |
| --- | --- | --- | --- |
| **Articles checked (record number):** | | | **Library:** |
| **Total number of articles:** | | | **Custom Group:** |
| **Date:** | | | **Sorted by:** |
| **The excluded articles do not live up to at least one of the criteria.** | | | **Number of articles:** |
| **1-4 Formalia** | | |  |
| **1** |  | Language; Swedish, English, Finnish |  |
| **2** |  | Peer reviewed primary research articles, published in scientific journals (excl. books) |  |
| **3** |  | Years of publication; 1995-2016 |  |
| **4** |  | Other reason for exclusion |  |
| **intr** |  | Interesting for discussion/introduction |  |
| **incl** |  | Include at this stage/too little information to exclude |  |
| **5-7 Population** | | |  |
| **5** |  | Studies using data on individuals living in the Nordic countries; Sweden, Norway, Finland, Denmark and Iceland. |  |
|  | ***b*** | *Excluding: Svalbard, the Faeroe Islands and Greenland.* |  |
| **6** |  | Studies that compare groups of humans, categorised by country of birth. |  |
|  | ***b*** | *Excludes: Studies groups not categorized by the individuals own country of birth.* |  |
|  | ***c*** | *Excludes: Studies which do not compare groups categorized by country of birth, with the majority population, within a country.* |  |
|  | ***d*** | *Excludes: Other minorities, not categorised by the individuals owns country of birth (ex. second generation immigrants).* |  |
|  | ***e*** | *Excludes: Studies not human beings, instead; countries, states, geography, animals, chemistry, biology (racemization etc.), ecological studies.* |  |
|  | ***f*** | *Excludes: Uses the terms on level one ("country of birth" etc.) to describe drop outs from their data or to describe their results, for example: "a minority of the observations…"* |  |
| **7** |  | Studies focused on working-age adult individuals, aged 18 or above. Or total population. |  |
| **8 Outcome** | | |  |
| **8** |  | Studies with all cause and/or specific causes of mortality among the primary outcomes, mentioned in the title or abstract. |  |
|  | ***b*** | *Excludes: studies only an intervention/healthcare/medicine (drugs)/measures/morbidity etc.* |  |
|  | ***c*** | *Excludes: studies mentioning mortality, but not as a primary outcome.* |  |
|  | ***d*** | *Excludes: Studies not reporting relative measures of mortality.* |  |

## S3. Reference list of included articles

1. Ferrada-Noli M, Asberg M, Ormstad K, Nordstrom P. Definite and undetermined forensic diagnoses of suicide among immigrants in Sweden. Acta Psychiat Scand. 1995;91(2):130-135.
2. Ferrada-Noli M. A cross-cultural breakdown of Swedish suicide. Acta Psychiat Scand. 1997;96(2):108-116.
3. Ferrada-Noli M, Asberg M. Psychiatric health, ethnicity and socioeconomic factors among suicides in Stockholm. Psychol Rep. 1997;81(1):323-332.
4. Johansson LM, Johansson SE, Bergman B, Sundquist J. Suicide, ethnicity and psychiatric in-patient care: A case-control study. Arc Suicide Res. 1997;3(4):253-269.
5. Johansson LM, Sundquist J, Johansson SE, Bergman B. Ethnicity, social factors, illness and suicide: a follow-up study of a random sample of the Swedish population. Acta Psychiat Scand. 1997;95(2):125-131.
6. Johansson LM, Sundquist J, Johansson SE, Bergman B, Qvist J, Traskman-Bendz L. Suicide among foreign-born minorities and native Swedes: An epidemiological follow-up study of a defined population. Soc Sci Med. 1997;44(2):181-187.
7. Johansson LM, Sundquist J. The influence of ethnicity and social and demographic factors on Swedish suicide rates. A four year follow-up study. Soc Psychiatry Psychiatr Epidemiol. 1997;32(3):165-170.
8. Sundquist J, Johansson SE. The influence of country of birth on mortality from all causes and cardiovascular disease in Sweden 1979-1993. Int J Epidemiol. 1997;26(2):279-287.
9. Bayard-Burfield L, Sundquist J, Johansson SE. Self-reported long-standing psychiatric illness as a predictor of premature all-cause mortality and violent death: a 14-year follow-up study of native Swedes and foreign-born migrants. Soc Psychiatry Psychiatr Epidemiol. 1998;33(10):491-496.
10. Weitoft GR, Gullberg A, Hjern A, Rosen M. Mortality statistics in immigrant research: method for adjusting underestimation of mortality. Int J Epidemiol. 1999;28(4):756-763.
11. Allgulander C, Nilsson B. Victims of criminal homicide in Sweden: a matched case-control study of health and social risk factors among all 1,739 cases during 1978-1994. Am J Psychiatry. 2000;157(2):244-247.
12. Hjern A, Allebeck P. Suicide in first- and second-generation immigrants in Sweden: a comparative study. Soc Psychiatry Psychiatr Epidemiol. 2002;37(9):423-429.
13. Pudaric S, Sundquist J, Johansson S-E. Country of birth, instrumental activities of daily living, self-rated health and mortality: A Swedish population-based survey of people aged 55-74. Soc Sci Med. 2003;56(12):2493-2503.
14. Albin B, Hjelm K, Ekberg J, Elmstahl S. Mortality among 723,948 foreign- and native-born Swedes 1970-1999. Eur J Public Health. 2005;15(5):511-517.
15. Sundaram V, Qin P, Zollner L. Suicide risk among persons with foreign background in Denmark. Suicide Life Threat Behav. 2006;36(4):481-489.
16. Westman J, Sundquist J, Johansson LM, Johansson SE, Sundquist K. Country of birth and suicide: a follow-up study of a national cohort in Sweden. Arch Suicide Res. 2006;10(3):239-248.
17. Hedlund E, Pehrsson K, Lange A, Hammar N. Country of birth and survival after a first myocardial infarction in Stockholm, Sweden. Eur J Epidemiol. 2008;23(5):341-347.
18. Klinthäll M, Lindstrom M. Migration and health: a study of effects of early life experiences and current socio-economic situation on mortality of immigrants in Sweden. Ethn Health. 2011;16(6):601-623.
19. Beiki O, Hall P, Ekbom A, Moradi T. Breast cancer incidence and case fatality among 4.7 million women in relation to social and ethnic background: a population-based cohort study. Breast Cancer Res. 2012;14(1):R5.
20. Borne Y, Engstrom G, Essen B, Hedblad B. Immigrant status and increased risk of heart failure: the role of hypertension and life-style risk factors. BMC Cardiovasc Disord. 2012;12:20.
21. Johansson B, Helgesson M, Lundberg I, Nordquist T, Leijon O, Lindberg P, Vingard E. Work and health among immigrants and native Swedes 1990-2008: a register-based study on hospitalization for common potentially work-related disorders, disability pension and mortality. BMC Public Health. 2012;12:845.
22. Li X, Sundquist K, Sundquist J. Neighborhood deprivation and prostate cancer mortality: A multilevel analysis from Sweden. Prostate Cancer Prostatic Dis. 2012;15(2):128-134.
23. Mousavi SM, Sundquist K, Hemminki K. Morbidity and mortality in gynecological cancers among first- and second-generation immigrants in Sweden. Int J Cancer. 2012;131(2):497-504.
24. Norredam M, Olsbjerg M, Petersen JH, Bygbjerg I, Krasnik A. Mortality from infectious diseases among refugees and immigrants compared to native Danes: a historical prospective cohort study. Trop Med Int Health. 2012;17(2):223-230.
25. Norredam M, Olsbjerg M, Petersen JH, Juel K, Krasnik A. Inequalities in mortality among refugees and immigrants compared to native Danes--a historical prospective cohort study. BMC Public Health. 2012;12:757.
26. Spallek J, Arnold M, Razum O, Juel K, Rey G, Deboosere P, Mackenbach JP, Kunst AE. Cancer mortality patterns among Turkish immigrants in four European countries and in Turkey. Eur J Epidemiol. 2012;27(12):915-921.
27. Esscher A, Haglund B, Hogberg U, Essen B. Excess mortality in women of reproductive age from low-income countries: a Swedish national register study. Eur J Public Health. 2013;23(2):274-279.
28. Hemminki K, Ankerst DP, Sundquist J, Mousavi SM. Prostate cancer incidence and survival in immigrants to Sweden. World J Urol. 2013;31(6):1483-1488.
29. Mousavi SM, Forsti A, Sundquist J, Hemminki K. Ethnic differences in breast cancer risk and survival: a study on immigrants in Sweden. Acta Oncol. 2013;52(8):1637-1642.
30. Norredam M, Olsbjerg M, Petersen JH, Laursen B, Krasnik A. Are there differences in injury mortality among refugees and immigrants compared with native-born? Inj Prev. 2013;19(2):100-105.
31. Rafnsson SB, Bhopal RS, Agyemang C, Fagot-Campagna A, Harding S, Hammar N, Hedlund E, Juel K, Primatesta P, Rosato M et al. Sizable variations in circulatory disease mortality by region and country of birth in six European countries. Eur J Public Health. 2013;23(4):594-605.
32. Abdoli G, Bottai M, Moradi T. Cancer mortality by country of birth, sex, and socioeconomic position in Sweden, 1961-2009. PLoS One. 2014;9(3):e93174.
33. Albin B, Hjelm K, Elmstahl S. Comparison of stroke mortality in Finnish-born migrants living in Sweden 1970-1999 and in Swedish-born individuals. J Immigr Minor Health. 2014;16(1):18-23.
34. Lindmark A, Glader EL, Asplund K, Norrving B, Eriksson M. Socioeconomic disparities in stroke case fatality--Observations from Riks-Stroke, the Swedish stroke register. Int J Stroke. 2014;9(4):429-436.
35. Norredam M, Olsbjerg M, Petersen JH, Hutchings M, Krasnik A. Cancer mortality does not differ between migrants and Danish-born patients. Dan Med J. 2014;61(6):A4848.
36. Rostila M, Fritzell J. Mortality differentials by immigrant groups in Sweden: the contribution of socioeconomic position. Am J Public Health. 2014;104(4):686-695.
37. Abdoli G, Bottai M, Sidorchuk A, Moradi T. Trends in mortality after cancer diagnosis: A nationwide cohort study over 45 years of follow-up in Sweden by country of birth. Cancer Epidemiol. 2015;39(4):633-640.
38. Di Thiene D, Alexanderson K, Tinghog P, La Torre G, Mittendorfer-Rutz E. Suicide among first-generation and second-generation immigrants in Sweden: association with labour market marginalisation and morbidity. J Epidemiol Community Health. 2015;69(5):467-473.
39. Ikram UZ, Malmusi D, Juel K, Rey G, Kunst AE. Association between Integration Policies and Immigrants' Mortality: An Explorative Study across Three European Countries. PLoS One. 2015;10(6):e0129916.
40. Li X, Sundquist J, Zoller B, Sundquist K. Neighborhood deprivation and lung cancer incidence and mortality: a multilevel analysis from Sweden. J Thorac Oncol. 2015;10(2):256-263.
41. Santosa A, Rocklöv J, Högberg U, Byass P. Achieving a 25% reduction in premature non-communicable disease mortality: The Swedish population as a cohort study. BMC Med. 2015;13(1).
42. Simberg-Danell C, Lyth J, Mansson-Brahme E, Frohm-Nilsson M, Carstensen J, Hansson J, Eriksson H. Prognostic factors and disease-specific survival among immigrants diagnosed with cutaneous malignant melanoma in Sweden. Int J Cancer. 2016;139(3):543-553.
43. Syse A, Strand BH, Naess O, Steingrimsdottir OA, Kumar BN. Differences in all-cause mortality: A comparison between immigrants and the host population in Norway 1990-2012. Demogr Res. 2016;34:615-655.

## S4. Results from studies analysing all-cause mortality in Sweden, for partly overlapping gender and country subgroups*

| **All-Cause Mortality** | **Studies with subgroups found in the respective "risk-category"** | | | | | |
| --- | --- | --- | --- | --- | --- | --- |
|  | **Significant**  **Increased** | **Uncertain Increase** | **No difference** | **Uncertain Decrease** | **Significant**  **Decreased** | **Studies** |
| Foreign-born | 21M, 41 | 9WM, 17M | 21W | 17W, 22 | - | 9, 17, 21, 22, 41 |
| ***Nordic*** | 10WM, 17M | - | - | 17W | - | 10, 17 |
| Finland | 8M, 9M, 14WM, 18M, 36WM | 8W, 17M, 18W | - | 17W | 13 | 8, 9, 13, 14, 17, 18, 36 |
| Norway | 18WM | 36W | 36M | - | - | 18, 36 |
| Denmark | 14WM, 18WM | 36W | - | 36M | - | 14, 18, 36 |
| ***Central Europe*** |  |  |  |  |  |  |
| Germany | 14M | - | 14W | 18WM, 36M | 36W | 14, 18, 36 |
| ***Eastern Europe*** | 10M | 8W | - | 8M, 10W, 17WM | 13 | 8, 10, 13, 17 |
| Poland | 14WM | - | - | - | 36WM | 14, 36 |
| ***Southern Europe*** | - | 8M | - | 8W | 10WM, 13 | 8, 10, 13 |
| F. Yugoslavia | 14M | 14W | - | 18WM | 36WM | 14, 18, 36 |
| ***Western countries*** | - | 8WM | - | - | 13 | 8, 13 |
| ***Latin America*** | - | 17 | - | - | 10WM | 10, 17 |

Only countries and country groupings with results in at least two studies are presented in this table

*Results from each study may appear in several categories

## S5. Results from studies analysing suicide and undetermined death in Sweden, for partly overlapping gender and country subgroups*

| **Suicide Mortality** | **Studies with subgroups found in the respective "risk-category"** | | | | | |
| --- | --- | --- | --- | --- | --- | --- |
|  | **Significant**  **Increased** | **Uncertain Increase** | **No difference** | **Uncertain Decrease** | **Significant**  **Decreased** | **Studies** |
| Foreign-born | 1, 2, 3, 5, 6WM, 7WM | 3, 4, 7WM, 9 | - | - | - | 1, 2, 3, 4, 5, 6, 7, 9 |
| ***Nordic*** |  |  |  |  |  |  |
| Finland | 1, 2, 6WM, 12, 16M | 16W | - | - | - | 1, 2, 6, 12, 16 |
| Norway | 2 | 1, 6M | - | 6W | - | 1, 2, 6 |
| Denmark | 2 | 1, 6WM | - | - | - | 1, 2, 6 |
| ***Western Europe*** | - | 12 | - | 6 | - | 6, 12 |
| ***Central Europe*** |  |  |  |  |  |  |
| Germany | 2 | 1, 6W | - | 6M | - | 1, 2, 6 |
| ***Eastern Europe*** | 6, 16W | 12 | - | 16M | - | 6, 12, 16 |
| Poland | 6W, 16W | 2, 6M, 16M | - | - | - | 2, 6, 16 |
| Hungary | 6W | 2 | 6M | - | - | 2, 6 |
| ***Southern Europe*** | - | - | - | 16W | 6, 12, 16M | 6, 12, 16 |
| F. Yugoslavia | - | 1 | - | 2, 6W | 6M | 1, 2, 6 |
| ***Middle East*** | - | - | - | - | 12, 16WM | 12, 16 |
| ***Russia*** | 2, 6WM | - | - | - | - | 2, 6 |

Only countries and country groupings with results in at least two studies are presented in this table

*Results from each study appears in several categories
