# Supplementary material for: Mortality by country of birth in the Nordic countries – a systematic review of the literature
Source: BMC Public Health. 2017 May 25;17:511. doi: 10.1186/s12889-017-4447-9 (PMC5445314; doi:10.1186/s12889-017-4447-9)
Supplement: Supplementary file 2 — Summary of descriptive information for included studies (See Additional file 1: S3 for a reference list of studies included). (DOCX 50 kb) [file 12889_2017_4447_MOESM2_ESM.docx]

Additional file 2

Table S1. Summary of descriptive information for included studies (See Supplement 3 for a reference list of studies included)

| **Study** | **Year** | **Study Design** | **Measure** | **Mortality** | **Population** | **Follow-Up** | **Data Type** | **Country Groupings** | **Grouping Rationale** | **Quality** |
| --- | --- | --- | --- | --- | --- | --- | --- | --- | --- | --- |
| 1. | 1995 | Cross-sectional | Standardized Mortality Ratio | Suicide | Total population of Stockholm, **Sweden**, aged >15 in Södermanland and Gotland, 1989/1990 (n=1,947,123) | 1989-1990 | Register | (1) Foreign-born  (2) Finland, Norway, Denmark, Germany, Middle East and Asia, Poland, Former Yugoslavia, Other countries | Population size | Low  Chi-square tests, no control variables, no national data, excluded refugees from analysis, country groupings/ rationale |
| 2. | 1997 | Cross-sectional | Standardized Mortality Ratio | Suicide | Mean **Swedish** population between 1987-1991 (n=8,526,951) | 1987-1991 | Register | (1) Foreign-born  (2) Finland, Norway, Denmark, Germany, Poland, Former Yugoslavia, Former Soviet Union, Iran, Hungary, Chile, USA, Former Czechoslovakia, Ethiopia, Austria, Korea, UK, Turkey, Italy, Spain, Netherlands, France, Iraq, Uruguay, Other countries | Population size | Medium  Chi-square tests, no control variables, total population, excluded refugees from analysis, country groupings/ rationale |
| 3. | 1997 | Cross-Sectional | χ^2^ Tests | Suicide | Suicide cases in Stockholm County, **Sweden** (n=1,722) within the total population (n=1,635,650) | 1987-1990 | Register | Foreign-born | No info | Low  Chi-square tests, no control variables, no national data, no specific country groupings |
| 4. | 1997 | Case-control | Odds Ratio | Suicide/ Undetermined death | All cases of psychiatric in-patients, aged 20-79, committed suicide in Oct. 1984-Oct. 1985, in Stockholm, **Sweden** Southern health care area (n=59); controls (n=630) | 1984-1986 | Register, Medical review | Foreign-born | No info | Low  Unconditional logistic regression, no control variables, no national data, specific subsample, no specific country groupings |
| 5. | 1997 | Cohort | Risk Ratio | Suicide/ Undetermined death | 7 independent, random samples of the **Swedish** population, aged 16-84, in 1979-1985 (n=52,169) | 1979-1993 | Register, Survey | Foreign-born | No info | High  Proportional hazards model, controls, total population, no specific country groupings |
| 6. | 1997 | Cohort | Risk Ratio | Suicide/ Undetermined death | Total population of individuals aged >15 in **Sweden** in 1985 (n=6,725,274) | 1985-1989 | Register | (1) Foreign-born  (2) Finland, Western Europe, Southern Europe, Eastern Europe, Other countries  (3) Finland, Norway, Denmark, Yugoslavia, Poland, Russia, Germany, Hungary | Culture, standard of living, population size | High  Poisson regression, controls, total population, country groupings/rationale |
| 7. | 1997 | Cohort | Rate Ratio | Suicide/ Undetermined death | All individuals aged >20 in **Sweden** in 1985 (n=6,283,099) | 1985-1989 | Register | Foreign-born | No info | High  Poisson regression, controls, total population, no specific country groupings |
| 8. | 1997 | Cohort | Relative Risk | Various | 7 independent, random samples of the **Swedish** population, aged 20-74 (n=43,397) | 1975-1993 | Register, Survey | Finland, Western countries, Southern Europe, Eastern Europe, Other countries | Standard of living, culture, geography | Medium  Proportional hazards model, controls, random samples, country groupings/rationale |
| 9. | 1998 | Cohort | Hazard Ratio | Various | 7 independent, random samples of the **Swedish** population, aged 25-74 (n=39,155 total) | 1979-1996 | Register, Survey | (1) Foreign-born  (2) Finnish, Other foreign-born | No info | Medium  Proportional hazards model, controls, random samples of total population, no grouping rationale |
| 10. | 1999 | Cohort | Age- Standardized Relative Risk | All-cause | All nationally registered individuals living in **Sweden** 1985-1994, aged 20-64 (n=9,856,240) | 1987-1994 | Register | Nordic countries, Eastern Europe, Southern Europe (and former Yugoslavia, Turkey), Rest of Europe/USA/Canada/ Oceania, Latin America, Africa and Asia | Systems for reporting cause of death, incentive to stay, type of migration | High  Controls, total population, country groupings/rationale |
| 11. | 2000 | Case-control | Relative Risk | Homicide | All cases of homicide in 1978-1994, aged 18+, living in **Sweden** (n=1,739) and matched controls (n=10,434) | 1977-1994 | Register | Denmark/Iceland/Norway, Non-Nordic, Finland | No info | Medium  Conditional logistic regression, no controls |
| 12. | 2002 | Cohort | Odds Ratio | Suicide | **Swedish** or foreign-born parents, born between 1929-1965 (n=1,470,000) | 1990-1998 | Register | Finland, Western Europe, Eastern Europe, Southern Europe, Middle East, Other non-European | Geography | High  Cox regression, controls, total population, country groupings/rationale |
| 13. | 2003 | Cohort | Risk Ratio | All-cause | 6 random samples of the **Swedish** population between 1986-1991, individuals aged 55-74 (n=8,959) | 1986-1998 | Register, Survey | Finland, Western countries, Southern Europe, Eastern European, Developing countries | Geography, duration of stay, residence permit | High  Proportional hazards model, controls, random samples of total population, country groupings/rationale |
| 14. | 2005 | Case-control | Odds Ratio | All-cause | Foreign-born persons, aged >16, registered living in **Sweden** (n=361,974) and matched native Swedish controls (n=361,974) | 1970-1999 | Register | Denmark, Finland, Norway/Iceland, Yugoslavia, Poland, Germany, Other European countries, Non-European countries, Stateless/unknown | No info | Medium  Chi-square tests, no controls, total population, no grouping rationale |
| 15. | 2006 | Case-control | Odds Ratio/ Incidence Risk Ratio | Suicide | All cases of suicide in **Denmark** between 1981-1997, aged <45 (n=8,137) and matched controls (n=162,740) | 1981-1997 | Register | "Foreign-born with at least one Danish-born parent", "Foreign-born with only foreign-born parent(s)", Nordic, Western Europe, Eastern Europe, Asia, Africa/Other | No info | Medium  Conditional logistic regression, controls, total population, no grouping rationale |
| 16. | 2006 | Cohort | Hazard Ratio | Suicide | All individuals with a personal identification number living in **Sweden** in 1994, aged 25-64 (n=4.4 mil) | 1994-1999 | Register | Finland, Southern Europe, OECD countries, Poland, Eastern Europe, Middle East, Other non-European countries | Geography, economic/ cultural similarities, population size | High  Cox regression, controls, total population, country groupings/rationale |
| 17. | 2008 | Cohort | Hazard Ratio | All-cause | All survivors (of 28 days) treated for a first myocardial infarction in Stockholm, **Sweden** between 1985-1997, aged 30-74 (n=>22,000) | 1985-1997 | Register | (1) Foreign-born (2) Foreign-born except born in Finland (3) Finland, Other Nordic, Baltic, Eastern Europe, Western Europe, Middle East, Latin America, Asia, Africa | Geography | Low  Cox regression, controls, no national data, specific subsample, country groupings/rationale |
| 18. | 2011 | Cohort | Hazard Ratio | Various | Random sample of **Swedish** residents born between 1921-1939, women (n=16,022) and men (n=18,673) | 1980-2001 | Register | Chile, Czechoslovakia, Denmark,  Finland, Germany, Greece, Italy, Norway, USA, Former Yugoslavia | Infant mortality/ GDP per capita info available | High  Cox regression, controls, random samples of total population, country groupings/rationale |
| 19. | 2012 | Cohort | Hazard Ratio | Breast cancer | All women living in **Sweden** from 1961, including immigrants (n=760,214) and natives (n=3,297,353) | 1961-2007 | Register | (1) Foreign-born  (2) Africa, Asia, Europe, Latin America, North America, Oceania  (3) Africa (Eastern/Middle, Northern, Southern, Western), Asia (Eastern, South-Central, South-Eastern, Western), Europe (Eastern, Northern, Southern, Western)  (4) Africa (Ethiopia, Other Eastern/ Middle), Asia (India, Iran, Other South-Central, Thailand, Other South-Eastern), Iraq, Lebanon, Syria, Turkey, Other Western), Europe (Czechoslovakia, Hungary, Poland, Romania, Soviet Union, Other Eastern, Denmark, Estonia, Finland, Iceland, Latvia, Norway, UK, Bosnia, Greece, Italy, Portugal, Spain, Yugoslavia, Austria, France, Germany, Netherlands, Other Western), Latin America (Chile, Uruguay, Other), North America (Canada, USA), Oceania | No info | High  Cox regression, controls, total population, no grouping rationale |
| 20. | 2012 | Cohort | Hazard Ratio | Heart failure | Subjects from Malmö, **Sweden** without history of myocardial infarction, stroke or heart failure, aged 45-73 (n=26,559) | 1991-2006 | Register | Foreign-born | No info | Low  Cox regression, controls, no national data, specific subsample, no specific country groupings |
| 21. | 2012 | Case-control | Hazard Ratio | All-cause | Legally immigrated persons in **Sweden** (n=243,860) and random age-matched Swedish-born aged >15 (n=859,653) | 1991-2008 | Register | Foreign-born | No info | Medium  Cox regression, controls, total population, no specific country groupings |
| 22. | 2012 | Cohort | Age-Standardized Mortality Rate | Various | **Swedish** male prostate cancer patients aged 25-74 (n=73,159) | 1990-2008 | Register | Foreign-born | No info | Medium  Multilevel logistic regression, controls, specific subsample, no specific country groupings |
| 23. | 2012 | Cohort | Standardized Mortality Ratio | Various | Cohort of natives (n=4,460,352) and first-generation immigrants (n=674,591) in **Sweden** | 1958-2008 | Register | (1) Foreign-born  (2) Finland, Denmark, Norway, Baltic country, Germany, Benelux, UK, Poland, Russia, Former Yugoslavia, Other Eastern Europe, Greece, Southern Europe, Other Europe, Turkey, Iraq, Iran, Asian Arab countries, Indian Subcontinent, Asia (Southeast, East, Other), North America, Chile, Latin America, Africa | Geography, population size | Medium  Ratio observed/expected deaths, controls, total population, country groupings/rationale |
| 24. | 2012 | Case-control | Hazard Ratio | Infectious disease | Refugees (n=29,139) and family-reunited immigrants (n=27,134) aged 18+, 4:1, age-sex matched with natives (n=116,556; 108,534) in **Denmark** | 1994-2007 | Register | Asia, Eastern Europe, Former Yugoslavia, Iraq, Middle East, North Africa, Sub-Saharan Africa | Geography, population size | High  Cox regression, controls, refugees and immigrants, country groupings/rationale |
| 25. | 2012 |  |  | Various |  | 1994-2007/08 |  |  |  |  |
| 26. | 2012 | Cohort | Mortality Rate | Cancer | Turkish immigrants (men, 182,399 person-years at risk; women, 162,014 PYR) and natives (men, 25,996,864 PYR; women, 26,655,302 PYR) in **Denmark** | 1992-2001 | Register | Turkey | No info | Medium  Poisson regression, controls, no sample size, one country-of-birth |
| 27. | 2013 | Cohort | Relative Risk | Various | Women of reproductive age (aged 15-49) living in **Sweden** (n=27,957) | 1988-2007 | Register | Low-income countries, Middle-income countries, High-income countries  (*See article for country-specific classifications*) | World Bank Class. of Economies | Medium  Poisson regression, no controls, subsample of national data, large country groupings/rationale |
| 28. | 2013 | Cohort | Hazard Ratio | Prostate cancer | Prostate cancer cases among natives (n=148,941) and foreign-born immigrants (n=7,872) in **Sweden** | 1958-2008 | Register | (1) Foreign-born  (2) Low-risk Europeans (Finland, Denmark, Norway, Germany, Benelux, Britain, Poland, East and South Europe, Russia, Former Yugoslavia, Greece), Low-risk non-Europeans (India, Latin America, Other Africa, Other), Very low-risk non-Europeans (Turkey, Iraq, Iran, Asian Arabic countries, Southeast and East Asia, Chile, North Africa), Other (Baltics, North America) | Geography, prostate cancer risk | High  Cox regression, controls, national data, specific subsample, large country groupings/rationale |
| 29. | 2013 | Cohort | Hazard Ratio | Breast cancer | Breast cancer cases among natives (n=137,547) and foreign-born immigrants (n=12,505) in **Sweden** | 1958-2008 | Register | (1) Foreign-born  (2) High-risk countries (Iraq), Low-risk Europeans (Baltic countries, Greece), Low-risk non-Europeans (India, East Asia, Latin America, Other Africa), Lowest-risk non-Europeans (Turkey, Southeast Asia, Chile), Other (Finland, Denmark, Norway, Germany, Benelux, Britain, Poland, East and South Europe, Russia, Former Yugoslavia, Iran, Asian Arabic countries, North America, North Africa, Iceland, Andorra, Cyprus, Israel, Malta, Monaco, Portugal, San Marino, State of Vatican City, Albania, Macedonia, Moldova, Slovenia, Afghanistan, Armenia, Azerbaijan, Georgia, Kazakhstan, Kyrgyzstan, Tajikistan, Turkmenistan, Ukraine, Uzbekistan, Belarus) | Geography, breast cancer risk | High  Cox regression, controls, national data, specific subsample, large country groupings/rationale |
| 30. | 2013 | Case-control | Rate Ratio | Injury | Refugees (n=29,139) and family reunited immigrants (n=27,134) matched with native controls (n=116,556 and 108,534, respectively) in **Denmark** | 1994-2007 | Register | (1) Foreign-born  (2) Only significant results from: Former Yugoslavia, Iraq, Middle East, Sub-Saharan Africa | No info | Medium  Cox regression, controls, refugees and immigrants, no grouping rationale, selective reporting of results |
| 31. | 2013 | Cohort | Mortality Rate Ratio | Various | **Danish** residents aged 35-74, including natives (men, 12,341,982 person-years at risk; women, 12,546,253 PYR) and immigrants (men, 196,786 PYR; women, 187,394 PYR) | 1992-2001 | Register | South Asia (Afghanistan. Pakistan, Sri Lanka), Southeast Asia (Thailand, Vietnam), Eastern Europe (Bosnia/Herzegovina, Poland, Yugoslavia), Middle East (Iran, Iraq, Lebanon, Turkey) | Socio-economic deprivation, population size, data availability | Medium  Poisson log-linear model, controls, total population, variable denominators (Swedish results), country groupings/rationale |
|  |  |  |  |  | Residents of **Sweden** aged 35-74, including natives (men, 28,982,698 PYR; women, 28,925,653 PYR) and immigrants (men, 1,935,841 PYR; women, 2,121,537 PYR) | 1889-2006 | Register | East Asia (China, North/South Korea), South Asia (India), Southeast Asia (Thailand, Vietnam), Eastern Europe (Poland, Former Yugoslavia, Former USSR European, Baltic States, Former USSR Eastern, Eastern Europe), Southern Latin America (Chile), Middle East (Iraq, Iran, Lebanon, Turkey), East Sub-Saharan Africa (Ethiopia, Eretria, Somalia) |  |  |
| 32. | 2014 | Cohort | Mortality Rate Ratio | Cancer | Total **Swedish** population (men, n=7,109,327; women, n=6,958,714) | 1961-2009 | Register | (1) Foreign-born  (2) Africa (East, Central, North, Southern, and West Africa), Asia (East, South-Central, South-East, and Western Asia), Europe (Eastern, Northern, Southern, and Western Europe), Latin America (Caribbean, Central America, and South America), North America, and Oceania (Australia/New Zealand, Melanesia, and Micronesia/Polynesia)  (3) *See paper for countries* | United Nations Population Division, population size | High  Poisson regression, controls, total population, country groupings/rationale |
| 33. | 2014 | Case-control | Odds Ratio | Stroke | Population sample in **Sweden** aged 16+ including native controls (men, n=53,062; women, n=68,218) and Finnish-born (men, n=53,062; women, n=68,218) | 1970-1979  1980-1989  1990-1999 | Register | Finland | Evidence of increased mortality | Medium  Logistic regression, no controls, stratify by study period, one country-of-birth/rationale |
| 34. | 2014 | Cohort | Odds Ratio | Stroke | Riks-Stroke sample of patients aged 18-74 (n=62,497) in **Sweden** | 2001-2009 | Register | Nordic countries (Norway, Finland, Denmark, Iceland), Europe (excluding Nordic countries), Other countries | No info | Medium  Multiple logistic regression, controls, specific subsample, no grouping rationale |
| 35. | 2014 | Case-control | Hazard Ratio | Cancer | Non-Western migrants in **Denmark** aged 18+ who arrived between 1993-1999 (n=56,723), age- and sex-matched 1:4 with natives (n=225,090) | 1994-2007 | Register | Eastern Europe (incl. Former Yugoslavia), Middle East (incl. North Africa), Other non-Western countries | Geography, population size | High  Cox regression, controls, specific subsample, country groupings/rationale |
| 36. | 2014 | Cohort | Hazard Ratio | Various | Total **Swedish** population aged 30-65 (men, n=1,997,666; women, n=1,964,965) | 1998-2006 | Register | Finland, Iraq, Former Yugoslavia, Poland, Iran, Bosnia, Germany, Denmark, Norway, Turkey, Somalia, Thailand | Population size | High  Cox regression, controls, total population, country groupings/rationale |
| 37. | 2015 | Cohort | Hazard Ratio | Cancer | Residents of **Sweden** diagnosed with cancer and aged 45+ (n=1,556,108; including 109,915 foreign-born) | 1961-2009 | Register | Foreign-born | No info | Medium  Cox regression, controls, specific subsample, results stratified by various factors, no specific country groupings |
| 38. | 2015 | Cohort | Hazard Ratio | Suicide | Total **Swedish** population aged 16-50 (n=4,034,728) | 2005-2010 | Register | Other Nordic countries (Finland, Denmark, Norway, Iceland), EU-25 and Other Western countries (EU in 2006, USA, Canada, Australia, New Zealand), Europe Outside EU, Rest | Geography | High  Cox regression, controls, total population, total population, country groupings/rationale |
| 39. | 2015 | Cohort | Mortality Rate Ratio | Various | Native-born (men, 17,369,353 person-years at risk; women, 17,103,404 PYR), Turkish-born (men, 105,719 PYR; women, 88,042 PYR) and Moroccan-born (men, 21,442 PYR; women, 14,794 PYR) residents of **Denmark** aged 20-69 | 1992-2001 | Register | Turkey, Morocco | No info | Medium  Poisson regression, controls, specific countries-of-birth, no grouping rationale |
| 40. | 2015 | Cohort | Odds Ratio | Lung cancer | Total **Swedish** population aged >50 (n=3.2 mil) | 2000-2010 | Register | Foreign-born | No info | Medium  Multilevel logistic regression, controls, total population, no specific country groupings |
| 41. | 2015 | Cohort | Mortality Rate Ratio | Various | Total **Swedish** population aged 30-69 (70,768,848 person-years) | 1991-2006 | Register | Foreign-born | No info | Medium  Poisson regression, controls, total population, reversed reference groups |
| 42. | 2016 | Cohort | Hazard Ratio | Cutaneous malignant melanoma | Population-based sample of cutaneous malignant melanoma cases (n=27,235; including 1,865 first-generation immigrants) in **Sweden** | 1990-2013 | Register | (1) Foreign-born  (2) Other Nordic countries, Western Europe, Eastern Europe, Southern Europe, Non-European countries, Former Yugoslavia | Geography | Medium  Cox regression, controls, specific subsample, country groupings/rationale |
| 43. | 2016 | Cohort | Odds Ratio | All-cause | Total **Norwegian** population aged 25-79 (n=4.4 mil) | 1990-2012 | Register | (1) Foreign-born  (2) Nordic, Europe (West, East), Middle East, South Asia, Asia, Africa, North America/Oceania, Other  (3) Somalia, Poland, Pakistan, Iran, Iraq, Vietnam, Thailand | Population size | High  Discrete time hazard regression, controls, total population, country groupings/rationale |
