# Supplementary material for: Mortality by country of birth in the Nordic countries – a systematic review of the literature
Source: BMC Public Health. 2017 May 25;17:511. doi: 10.1186/s12889-017-4447-9 (PMC5445314; doi:10.1186/s12889-017-4447-9)
Supplement: Supplementary file 3 — Summary of models and results for included studies (See Additional file 1: S3 for a reference list of studies included). (DOCX 102 kb) [file 12889_2017_4447_MOESM3_ESM.docx]

Additional file 3

Table S2. Summary of models and results for included studies (See Supplement 3 for a reference list of studies included)

| **Study** | **Mortality Outcome** | **Control Variables** | **Results according to authors** | **Excess risk** | **Uncertain increased risk/ Non-significant** | **No difference** | **Uncertain decreased risk/ Non-significant** | **Decreased risk** | **Reference category** | **No. of comparison groups** |
| --- | --- | --- | --- | --- | --- | --- | --- | --- | --- | --- |
| 1. | Suicide | - | “Immigrant status should be considered as a risk factor for suicide in Sweden." | *Reporting:*  *Standardized mortality ratios and p-values*  Foreign-born  1.52, p=0.003  Finland  2.51, p=0.002 | Norway  1.85, p=0.8 Denmark  2.18, p=0.1 Germany  2.16, p=0.2 Middle East/Asia  1.01, p=0.9 Former Yugoslavia  2.12, p=0.3 | - | Poland  0.99, p=0.9 Other countries  0.40, p=0.4 | - | Swedish | 1+8 |
| 2. | Suicide | - | "The risk of an immigrant dying of a cause related to suicide was found to be 1.5 times higher than that for a native Swede." | *Reporting:*  *Standardized mortality ratios and p-values*  Foreign-born  1.32, p=0.0001  Finland  2.23, p=0.0001 Norway  1.42, p=0.03 Denmark  1.50, p=0.02 Germany  1.70, p=0.004 Former Soviet Union  3.66, p=0.0002 | Poland  1.17, p=0.5 Hungary  1.52, p=0.09 Former Czechoslovakia 1.40, p=0.4 Ethiopia  1.40, p=0.4 Austria  1.50, p=0.4 Korea  1.11, p=0.08 Spain  1.16, p=0.8 Netherlands  1.50, p=0.5 France  1.50, p=0.5 Uruguay  1.66, p=0.3 | USA  1.0, p=1.0 | Former Yugoslavia 0.9 Iran  0.66 Chile  0.55 UK  0.69 Turkey  0.3 Italy  0.85 Iraq  0.45 Other countries 0.34 | - | Swedish | 1+24 |
| 3. | Suicide | - | "Both in low- and high-income areas, the incidence of suicide was higher among immigrants than among native Swedes." | *Reporting:*  *Chi-square tests and p-values*  Foreign-born  Low income area:  3.9, p=0.04 | Foreign-born  High income area:  0.4, p=0.5 | - | - | - | Swedish | 1 |
| 4. | Suicide and undetermined death | Sex  Age  Marital status  Somatic disease  Employment | "In the final model the odds ratio for foreign-born people decreased to 1.81, a non-significant tendency for suicide." | *Reporting:*  *Odds ratios and 95% confidence intervals* | Foreign-born  1.81 (0.78-4.20) | - | - | - | Swedish | 1 |
| 5. | Suicide and undetermined death | Sex  Age | "Ethnicity, defined as being born outside Sweden, had a risk ratio of 1.87 (1.118-2.97) in a model controlled for sex and age." | *Reporting:*  *Risk ratios and 95% confidence intervals*  Foreign-born  1.87 (1.118-2.97) | - | - | - | - | Swedish | 1 |
| 6. | Suicide and undetermined death | Age  Marital status (*total* *foreign-born only*)  Sex (*region-specific only*) | "Ethnicity, defined as being foreign-born, was a risk factor for suicide for both men and women… with control for age and marital status." | *Reporting:*  *Risk ratios and 95% confidence intervals*  Foreign-born WM  1.36 (1.21-1.53)  1.21 (1.11-1.31)  Regional  Finland  1.92 (1.75-2.10)  Eastern Europe  1.71 (1.42-2.06)  Country  Finland WM  1.68 (1.43-1.98)  2.04 (1.83-2.28)  Poland W  1.63 (1.04-2.56)  Russia WM  3.71 (2.05-6.71)  4.36 (2.89-6.58)  Hungary W  3.39 (2.04-5.63) | Country  Norway M  1.16 (0.83-1.62)  Denmark WM  1.50 (0.98-2.31)  1.05 (0.77 1.43)  Poland M  1.14 (0.72-1.79)  Germany W  1.44 (0.96-2.16) | Country  Hungary M  1.00 (0.59-1.69) | Regional  Western Europe  0.94 (0.82-1.08)  Country  Norway W  0.99 (0.63-1.50)  Yugoslavia W  0.48 (0.22-1.07)  Germany M  0.82 (0.55-1.21) | Regional  Southern Europe  0.50 (0.37-0.68)  Outside Europe  0.76 (0.61-0.96)  Country  Yugoslavia M  0.62 (0.41-0.94) | Swedish | 1+5+8 |
| 7. | Suicide and undetermined death | Marital status  Geographical region  Form of tenure  Overcrowding | "The main finding in this study was that ethnicity, defined as being foreign-born, was a significant risk factor for suicide in both sexes and in all age groups except for males aged 30 to 49 years." | *Reporting:*  *Risk ratios and 95% confidence intervals*  Foreign-born:  M age 20-29  1.43 (1.14-1.78)  M above 50  1.32 (1.14-1.53) W age 30-49  1.42 (1.19-1.71)  W above 50  1.62 (1.35-1.95) | Foreign-born: M aged 30-49  1.10 (0.97 1.24)  W aged 20-29  1.47 (0.96-2.17) | - | - | - | Swedish | 1 |
| 8. | (1) All-cause mortality  (2) Circulatory disease mortality  (3) Coronary heart disease mortality | Age  Marital status  Form of tenure  Years of education  Marital status*Age | "The increased mortality risk for Finnish males and females and, in addition to the increased circulatory disease mortality risk for Finnish females and the strongly increased risk for CHD mortality for females born in Finland and Eastern Europe could not be explained by confounding by age, marital status or socioeconomic position." | *Reporting:*  *Relative risks and 95% confidence intervals*  (1) All-cause  Finland M  1.51 (1.17-1.94)  (2) Circulatory  Finland W  2.15 (1.45-3.20)  (3) CHD  Finland W  2.18 (1.24-3.81)  East Europe W  3.02 (1.24–7.34) | (1) All-cause Finland W  1.29 (0.97-1.71) Western countries  WM  1.04 (0.78-1.38)  1.06 (0.83-1.34) South Europe M  1.34 (0.85-2.12) East Europe W  1.17 (0.66-2.06)  (2) Circulatory  Finland M  1.34 (0.90-1.99) Western countries W  1.08 (0.68-1.71)  East Europe W  1.76 (0.79-3.94) Other countries M  1.22 (0.61-2.45)  (3) CHD  Finland M  1.25 (0.76-2.06) | - | (1) All-cause South Europe W  0.73 (0.30-1.76) East Europe M  0.80 (0.48-1.33) Other countries WM  0.69 (0.31-1.55)  0.91 (0.53-1.53)  (2) Circulatory  West. countries M  0.87 (0.60-1.26) South Europe WM  0.49 (0.07-3.48)  0.83 (0.34-2.00) East Europe M  0.55 (0.23-1.32)  (3) CHD  Western countries WM  0.59 (0.24–1.43)  0.97 (0.64–1.49)  South Europe WM  0.99 (0.14–7.11)  0.97 (0.36–2.61)  East Europe M  0.64 (0.24–1.70)  Other countries M  0.69 (0.22–2.15) | - | Swedish | 5 |
| 9. | (1) All-cause mortality   (2) Suicides and accidents | Age  Long-standing psychiatric or somatic illness  No illness  Education  Civil status  Housing  Car ownership  Social network | "Only Finnish men demonstrated an independent increased all-cause mortality risk." | *Reporting:*  *Hazard ratios and 95% confidence intervals*  (1) All-cause  Finland M  1.41 (1.12-1.78) | (1) All-cause  Foreign-born WM  1.05 (0.89-1.24)  1.12 (0.97-1.29)  (2) Suicides and accidents  Foreign-born  1.06 (0.72-1.57) | - | - | - | Swedish | 2 |
| 10. | All-cause mortality | Age | "Significantly reduced death rates are found among immigrants outside the north-east of Europe." | *Reporting:*  *Standardized risk ratios and 95% confidence intervals*  Nordic WM  1.18 (1.14-1.23)  1.55 (1.51-1.60) Eastern Europe M  1.10 (1.03-1.18) | - | - | Eastern Europe W  0.99 (0.91-1.07) | South Europe WM  0.80 (0.72-0.88)  0.89 (0.84-0.95) Rest of Europe/ America WM  0.86 (0.78-0.94)  0.86 (0.80-0.92) Latin America WM  0.65 (0.52-0.81)  0.67 (0.56-0.81) Africa/Asia WM  0.75 (0.65-0.86)  0.70 (0.64-0.78) | Swedish | 6 |
| 11. | Homicide | Hospital diagnosis  Crime rate  Marital status | - | *Reporting:*  *Relative risk and 95% confidence intervals*  Non-Nordic WM  2.6 (1.7-4.0)  2.1 (1.4-3.0) Finland WM  2.5 (1.7-3.7)  3.9 (2.8-5.5) | Denmark/Iceland/Norway W  1.6 (0.8-3.4) | Denmark/Iceland/  Norway M  1.0 (0.5-2.0) | - | - | Swedish | 3 |
| 12. | Suicide | Birth year  Sex  SES  Single-parent household  Social welfare benefits  Housing  Geographic location of home | "In the multivariate analysis the Finnish and Western immigrants had high odds for suicide compared to the Swedish majority population... while the Middle-East immigrants had lower odds for suicide... The first-generation immigrants from Southern Europe had lower odds than the Swedish majority population." | *Reporting:*  *Odds ratios and 95% confidence intervals*  Finland  1.4 (1.2-1.7) | Eastern Europe  1.1 (0.7-1.5)  Western Europe  1.2 (1.0-1.6) | - | Outside Europe  0.6 (0.4-1.0) | Southern Europe  0.3 (0.2-0.5)  Middle East  0.3 (0.1-0.6) | Swedish | 6 |
| 13. | All-cause mortality | Age  Sex  Health status  Marital status  Social network  Education  Instrumental activities of daily living | "Country of birth was not associated with all-cause mortality." | *Reporting:*  *Risk ratios and 95% confidence intervals* | - | - | Finland  0.89 (0.64-1.22) Western countries  0.94 (0.72-1.22) Southern Europe  0.65 (0.27-1.57) Eastern Europe  0.79 (0.46-1.37) Developing countries  0.69 (0.26-1.85) | - | Swedish | 5 |
| 14. | All-cause mortality | - | "Migrants had higher mortality than the native population." | *Reporting:*  *Odds ratios and 95% confidence intervals*  Denmark WM  1.10 (1.07-1.125)  1.11 (1.08-1-13) Finland WM  1.08 (1.07-1.089)  1.21 (1.20-1.22) Iceland/Norway WM  1.04 (1.02-1.058)  1.07 (1.05-1.10) Yugoslavia M  1.04 (1.01-1.08) Poland WM  1.10 (1.06-1.44)  1.06 (1.02-1.11)  Germany M  1.04 (1.01-1.06) Other Europe M  1.05 (1.03-1.06) Outside Europe M  1.04 (1.02-1.07) | Yugoslavia W  1.03 (1.00-1.084)  Other Europe W  1.02 (1.00-1.04)  Outside Europe W  1.03 (1.00-1.05)  Stateless/  unknown WM  1.04 (0.87-1.25)  1.07 (0.87-1.31) | Germany W  1.00 (0.98-1.024) | - | - | Swedish | 9 |
| 15. | Suicide | Age  Sex  Calendar time Marital status  Annual income  Place of residence  Sickness  Job absence  Psychiatric history | "Suicide risk was generally higher among persons with foreign background compared with the majority population and the risk was highest among Nordic-born persons." | *Reporting:*  *Odds ratios and 95% confidence intervals*  Foreign-born/at least one Danish-born parent  1.77 (1.39–2.26) Nordic  2.40 (1.53–3.80) | Foreign-born/only foreign-born parent(s)  1.02 (0.77–1.36) Greenland  1.22 (0.91–1.62)  Western Europe/  North American  1.06 (0.82–1.36) | Eastern Europe  1.00 (0.73–1.35) | Africa/Other  0.85 (0.57–1.28) | Asia  0.61 (0.45–0.82) | Danish | 2+6 |
| 16. | Suicide | Age  Marital status  Socioeconomic status  Hospitalization for psychiatric disorder/  substance abuse | "Among men the highest risk of suicide was found among men from Finland. Among women the highest risk of suicide was found among women from Finland, Poland, and Eastern Europe." | *Reporting:*  *Hazard ratios and 95% confidence intervals*  OECD W  1.32 (1.02–1.71) Poland W  1.82 (1.27–2.61) East Europe W  1.50 (1.03–2.20) Finland M  1.16 (1.03–1.32) | Finland W  1.13 (0.94–1.36) OECD M  1.09 (0.90–1.31) Poland M  1.02 (0.64–1.61) | - | South Europe W  0.90 (0.57–1.44)  Outside Europe W  0.84 (0.54–1.29) East Europe M  0.83 (0.59–1.16) | Middle East WM  0.42 (0.23–0.78)  0.53 (0.41–0.69) South Europe M  0.72 (0.54– 0.97) Outside Europe M  0.66 (0.49–0.89) | Swedish | 7 |
| 17. | All-cause mortality  (*1-year mortality in survivors of 28 days after first myocardial infarction*) | Age  Calendar year  Socioeconomic status | "Immigrants in Sweden in general do not seem to have a higher mortality after a first myocardial infarction than Sweden-born, in particular when differences in socioeconomic status are accounted for. A higher CHD mortality in immigrants appears to be primarily due to an elevated disease incidence." | *Reporting:*  *Hazard ratios and 95% confidence intervals*  Country  Other Nordic except Finland M  1.88 (1.22–2.91) | Foreign-born M  1.13 (0.91–1.41)  Foreign-born except Finland M  1.07 (0.82–1.38)  Country  Finland M  1.27 (0.90–1.79) Baltics W  1.81 (0.67–4.87) West Europe W  1.23 (0.46–3.34) Middle East W  2.55 (0.35–18.42) Latin American  1.84 (0.59–5.77) | - | Foreign-born W  0.90 (0.61–1.34)  Foreign-born except Finland W  0.84 (0.49–1.45)  Country  Finland W  0.97 (0.58–1.62)  Other Nordic except Finland W  0.79 (0.29–2.12) Baltics M  0.91 (0.43–1.92) East Europe WM  0.20 (0.03–1.40)  0.89 (0.51–1.54) West Europe M  0.86 (0.51–1.47) Middle East M  0.59 (0.19–1.83) Asia M  0.59 (0.08–4.20) | - | Swedish | 1+1+9 |
| 18. | (1) All-cause mortality  (2) Cardio-vascular mortality  (3) Cancer mortality  (4) Other causes of mortality | Infant mortality rate at birth  GDP at birth  Birth cohort  Age  Civil status  Income  Education  Welfare | “Men born in Denmark, Finland and Norway as well as women born in Denmark and Norway display significantly higher hazard rate ratios of total mortality than men and women born in Sweden, respectively, in the final models including both the early life factors and current socio-economic position... In contrast, our study also shows that men born in Greece as well as women born in Greece and Chile display significantly lower hazard rate ratios of total mortality than men and women born in Sweden, respectively.” | *Reporting:*  *Hazard ratios and 95% confidence intervals*  (1) All-cause  Denmark WM  1.19 (0.97-1.46)  1.23 (1.07-1.41)  Finland M  1.46 (1.26-1.69)  Norway WM  1.19 (1.00-1.42)  1.26 (1.11-1.43)  (2) Cardiovascular  Denmark M  1.26 (1.02-1.56)  Finland WM  1.63 (1.12-2.37)  1.69 (1.35-2.12)  Norway M  1.29 (1.06-1.56)  (3) Cancer  Norway M  1.24 (0.99-1.56)  (4) Other causes  Czechoslovakia M  2.12 (1.03-4.34)  Denmark M  1.30 (0.98-1.73)  Finland M  1.88 (1.42-2.48)  F. Yugoslavia M  2.73 (1.33-5.63) | (1) All-cause  Czechoslovakia M  1.17 (0.80-1.70)  Finland W  1.06 (0.87-1.30)  USA M  1.03 (0.78-1.38)  (2) Cardiovascular  Czechoslovakia WM  1.08 (0.40-2.92)  1.18 (0.65-2.14)  Denmark W  1.22 (0.83-1.79)  Germany W  1.30 (0.75-2.25)  Italy M  1.01 (0.63-1.61)  Norway W  1.25 (0.91-1.72)  F. Yugoslavia W  1.06 (0.42-2.68)  (3) Cancer  Denmark WM  1.11 (0.82-1.51)  1.12 (0.87-1.43)  Norway W  1.11 (0.84-1.46)  USA W  1.13 (0.64-1.97)  (4) Other causes  Chile M  2.40 (0.57-10.2)  Denmark W  1.30 (0.88-1.90)  Finland W  1.12 (0.76-1.65)  Germany M  1.23 (0.80-1.91)  Italy M  1.03 (0.55-1.94)  Norway WM  1.25 (0.90-1.72)  1.22 (0.94-1.58)  USA M  1.53 (0.90-2.58)  F. Yugoslavia W  1.14 (0.44-2.96) | - | (1) All-cause  Chile M  0.61 (0.29-1.31)  Czechoslovakia W  0.85 (0.51-1.44)  Germany WM  0.98 (0.73-1.30)  0.95 (0.76-1.19)  Italy WM  0.95 (0.61-1.49)  0.97 (0.72-1.31)  USA W  0.83 (0.52-1.31)  F. Yugoslavia WM 0.69 (0.42-1.14)  0.98 (0.67-1.44)  (2) Cardiovascular  Chile WM  0.39 (0.06-2.59)  0.54 (0.16-1.79)  Germany M  0.93 (0.65-1.33)  Italy W  0.89 (0.37-2.15)  USA M  0.85 (0.52-1.39)  F. Yugoslavia M  0.84 (0.46-1.54)  (3) Cancer  Czechoslovakia WM  0.68 (0.31-1.49)  0.71 (0.37-1.36)  Finland M  0.93 (0.71-1.22)  Germany WM  0.84 (0.54-1.29)  0.77 (0.52-1.14)  Italy WM  0.92 (0.48-1.74)  0.81 (0.48-1.34)  USA M  0.93 (0.57-1.51)  (4) Other causes  Chile W  0.37 (0.05-2.62)  Czechoslovakia W  0.89 (0.32-2.48)  Germany W  0.89 (0.50-1.58)  Greece WM  0.99 (0.50-1.97)  0.85 (0.48-1.49)  Italy W  0.97 (0.39-2.40)  USA W  0.80 (0.31-2.01) | (1) All-cause  Chile W  0.27 (0.10-0.75)  Greece WM  0.46 (0.31-0.70)  0.60 (0.45-0.81)  (2) Cardiovascular  Greece WM  0.37 (0.16-0.86)  0.63 (0.41-0.99)  USA W  0.17 (0.02-1.20)  (3) Cancer  Chile WM  0.16 (0.03-0.75)  0.22 (0.06-0.86)  Finland W  0.73 (0.53-1.01)  Greece WM  0.27 (0.14-0.54)  0.43 (0.26-0.73)  F. Yugoslavia WM  0.35 (0.16-0.78)  0.47 (0.24-0.93) | Swedish | 10 |
| 19. | Breast cancer mortality | Follow-up age  Calendar period of diagnosis  Education  Residence at diagnosis | “When we stratified breast cancer case fatality by country of birth, we found a similar case fatality for most immigrants compared with native Swedes (Table 6). The risk of dying due to breast cancer, however, was 2.5 times higher among immigrants born in Northern Africa.” | *Reporting:*  *Hazard ratios and 95% confidence intervals*  *Note:*  *Country-specific findings not reported here*  Africa  Northern  2.81 (1.13-6.96) | Foreign-born  1.01 (0.95-1.07)  Africa  Total  1.10 (0.67-1.82)  Europe  Total   - 1. (0.95-1.08)   Total Eastern  1.05 (0.88-1.24)  Total Northern   - 1. (0.94-1.11)   Total Western  1.02 (0.86-1.21)  Latin America  Total  1.06 (0.67-1.66)  North America  Total  1.03 (0.49-2.16) | - | Africa  Total Eastern/ Middle  0.81 (0.43-1.52)  Southern  0.48 (0.05-4.23)  Asia  Total  0.91 (0.72-1.14)  Eastern  0.73 (0.31-1.74)  Total South-Central  0.93 (0.63-1.35)  Total South-Eastern  0.98 (0.49-1.98)  Total Western  0.91 (0.65-1.28)  Europe  Total Southern  0.95 (0.80-1.11)  Oceania  Total  0.45 (0.06-3.36) | - | Swedish | 1+58 |
| 20. | Heart failure mortality | Age  Sex  Year of HF hospitalization | “After adjustment for age, sex and year of the HF hospitalization, the immigrants group tended to have lower one-month and one-year mortality...” | *Reporting:*  *Hazard ratios and 95% confidence intervals* | - | - | One-month Foreign-born  0.20 (0.03-1.44)  One-year  Foreign-born  0.47 (0.22-1.01) | - | Swedish | 1 |
| 21. | All-cause mortality | Age  Education  Income  Years since first immigration | “The total immigrant cohort displayed increased HRs for almost all examined diagnoses and mortality… Adjustment for age, education, income, and years since first immigration, slightly lowered the immigrants’ initial HRs (Table 2).” | *Reporting:*  *Hazard ratios and 95% confidence intervals*  Foreign-born M  1.2 (1.1-1.2) | - | Foreign-born W  1.0 (0.9-1.0) | - | - | Swedish | 1 |
| 22. | (1) All-cause mortality  (2) Prostate cancer mortality | Neighborhood level  Age | - | *Reporting:*  *Odds ratios and 95% confidence intervals* | - | - | (1) All-cause  Foreign-born  0.98 (0.90-1.05)  (2) Prostate  Foreign-born  0.97 (0.88-1.07) | - | Swedish | 1 |
| 23. | (1) Cervical cancer mortality  (2) Endo-metrial cancer mortality  (3) Ovarian cancer mortality | 5-year age groups  10-year study time periods | “An increased cervical cancer mortality was seen among Danes (SMR = 1.77) followed by Norwegians (1.58).”  “Notably, first-generation immigrants had neither an increased endometrial cancer risk nor a significant difference in the mortality of this cancer.”  “The ovarian cancer mortality was decreased among Finns (SMR = 0.81), former Yugoslavians (0.69), Turks (0.25), Iranians (0.19), Chileans (0.23) and Latin Americans (0.41).” | *Reporting:*  *Standardized mortality ratios and 95% confidence intervals*  (1) Cervical  Denmark  1.77 (1.36-2.28)  Norway  1.58 (1.27- 1.94) | (1) Cervical  Foreign-born  1.02 (0.94-1.11)  Baltic country  1.37 (0.91-1.97)  Other East Europe  1.40 (0.93-2.04)  Other Europe  1.83 (0.59-4.26)  Indian Subcont.  1.84 (0.60-4.29)  Southeast Asia  1.09 (0.44-2.25)  (2) Endometrial  Benelux  1.60 (0.52-3.73)  Southern Europe  1.15 (0.46-2.37)  Other Europe  1.33 (0.16-4.81)  Iraq  1.03 (0.21-3.01)  Indian Subcont.  1.60 (0.19-5.79)  (3) Ovarian  Denmark  1.12 (0.90-1.37)  Norway  1.02 (0.86-1.21)  UK  1.30 (0.74-2.11)  Poland  1.01 (0.75-1.32) | (1) Cervical  Other Asia  1.00 (0.03-5.60) | (1) Cervical  Finland  0.93 (0.79-1.08)  Germany  0.80 (0.57-1.10)  Benelux  0.72 (0.15-2.11)  UK  0.74 (0.20-1.90)  Poland  0.85 (0.50-1.34)  Russia  0.65 (0.24-1.41)  Former Yugoslavia  0.96 (0.62-1.40)  Southern Europe  0.71 (0.26-1.54)  Turkey  0.48 (0.10-1.40)  East Asia  0.94 (0.25-2.40)  North America  0.92 (0.48-1.61)  Chile  0.77 (0.21-1.98)  Latin America  0.36 (0.04-1.30)  Africa  0.32 (0.04-1.15)  (2) Endometrial  Finland  0.88 (0.73-1.06)  Denmark  0.64 (0.38-1.02)  Norway  0.91 (0.66-1.23)  Baltic country  0.92 (0.53-1.47)  Germany  0.93 (0.66-1.28)  UK  0.55 (0.07-1.97)  Poland  0.96 (0.53-1.61)  Russia  0.65 (0.21-1.53)  Former Yugoslavia  0.68 (0.37-1.14)  Other East Europe  0.74 (0.37-1.32)  Greece  0.53 (0.06-1.93)  Turkey  0.44 (0.05-1.58)  Iran  0.24 (0.01-1.36)  Asian Arab c.  0.38 (0.01-2.10)  Southeast Asia  0.78 (0.09-2.80)  North America  0.94 (0.45-1.73)  Latin America  0.33 (0.01-1.87)  (3) Ovarian  Baltic country  0.83 (0.59-1.13)  Germany  0.96 (0.79-1.16)  Benelux  0.92 (0.42-1.75)  Russia  0.86 (0.52-1.35)  Other East Europe  0.96 (0.70-1.28)  Southern Europe  0.76 (0.42-1.25)  Other Europe  0.50 (0.10-1.47)  Iraq  0.59 (0.21-1.27)  Asian Arab c.  0.59 (0.22-1.29)  Indian Subcont.  0.38 (0.05-1.37)  Southeast Asia  0.60 (0.24-1.23)  East Asia  0.57 (0.18-1.32)  Other Asia  0.92 (0.11-3.33)  North America  0.71 (0.44-1.08)  Africa  0.44 (0.14-1.03) | (1) Cervical  Iran  0.14 (0.00-0.78)  (2) Endometrial  Foreign-born  0.83 (0.74-0.92)  (3) Ovarian  Foreign-born  0.83 (0.78-0.88)  Finland  0.81 (0.73-0.90)  Former Yugoslavia  0.69 (0.51-0.91)  Greece  0.07 (0.00-0.41)  Turkey  0.25 (0.07-0.65)  Iran  0.19 (0.04-0.54)  Chile  0.23 (0.05-0.68)  Latin America  0.41 (0.13-0.96) | Swedish | 1+26 |
| 24. | Infectious disease mortality | Age  Income | “Table 2 shows that overall, female refugees had a four-fold significantly increased mortality risk… compared with Danish born; male refugees had a twofold increased mortality risk.” | *Reporting:*  *Hazard ratios and 95% confidence intervals*  **Refugees**  Total WM  4.15 (2.38-7.25)  2.05 (1.27-3.33)  Asia M  5.04 (1.22- 20.80)  North Africa WM  18.43 (8.48-40.07)  9.58 (4.82-19.07)  Sub-Saharan Africa WM  195.7 (79.23-483.2)  8.11 (1.11-58.99) | F. Yugoslavia WM  1.54 (0.64-3.73)  1.40 (0.72-2.74))  Iraq W  2.35 (0.32-17.20) | - | Iraq M  0.51 (0.07-3.71)  Middle East M  0.72 (0.10-5.21) | - | Danish | 1+7 |
|  |  |  | “Overall, mortality risk did not differ significantly between female immigrants and their Danish-born control group… whereas for male immigrants, mortality risk was twice as high as for native men.” | **Immigrants**  Total M  2.40 (1.21- 4.79)  Eastern Europe M  7.73 (1.85-32.29)  Sub-Saharan Africa WM  22.48 (7.63-66.22)  10.25 (3.15-33.33) | Total W  1.23 (0.50-3.00)  Asia M  2.50 (0.60-10.42)  Middle East M  1.26 (0.30-5.25)  North Africa WM  2.90 (0.39-21.40)  1.64 (0.23-11.89) | - | Asia W  0.79 (0.11-5.82) | - | Danish | 1+7 |
| 25. | (1) All-cause mortality  (2) Cancer mortality  (3) Cardio-vascular mortality | Age  Income | “Overall, both refugee women… and men… had significantly lower all-cause mortality than native Danes.”  “Overall, refugee women… showed significantly lower mortality from cancer compared with native Danes, whereas the result for refugee men… was only borderline significant.”  “Overall, compared with native Danes, refugee women showed only border-line significant differences in mortality from CVD… while refugee men had a significantly lower mortality from CVD.” | *Reporting:*  *Hazard ratios and 95% confidence intervals*  **Refugees**  (1) All-cause  Sub-Saharan Africa W  2.70 (1.28 – 5.66) | (2) Cancer  *Refugees*  East Europe W  1.04 (0.33 – 3.22)  F. Yugoslavia M  1.01 (0.85 – 1.20)  Sub-Saharan Africa M  1.57 (0.39 – 6.28)  (3) Cardiovascular  Iraq W  1.31 (0.65 – 2.63) | - | (1) All-cause  Asia W  0.90 (0.50 – 1.63)  East Europe WM  0.75 (0.36 – 1.58)  0.59 (0.31 – 1.14)  North Africa WM  0.84 (0.61 – 1.16)  0.76 (0.58 – 1.00)  Sub-Saharan Africa M  0.52 (0.20 – 1.39)  (2) Cancer  Total M  0.86 (0.73 – 1.00)  Asia WM  0.50 (0.12 – 1.99)  0.15 (0.02 – 1.08)  East Europe M  0.32 (0.05 – 2.29)  F. Yugoslavia W  0.89 (0.75 – 1.07)  Middle East M  0.76 (0.49 – 1.19)  North Africa M  0.64 (0.32 – 1.28)  (3) Cardiovascular  Total W  0.80 (0.62 – 1.03)  Asia W  0.67 (0.09 – 4.75)  East Europe M  1.48 (0.47 – 4.60)  F. Yugoslavia W  0.83 (0.62 – 1.11)  Iraq M  0.68 (0.40 – 1.19)  Middle East W  0.78 (0.37 – 1.65)  North Africa WM  0.19 (0.03 – 1.32)  0.77 (0.35 – 1.73) | (1) All-cause  Total WM  0.78 (0.71 – 0.85)  0.64 (0.59 – 0.69)  Asia M  0.41 (0.24 – 0.71)  F. Yugoslavia WM  0.81 (0.73 – 0.90)  0.70 (0.63 – 0.77)  Iraq WM  0.62 (0.44 – 0.87)  0.45 (0.35 – 0.58)  Middle East WM  0.53 (0.38 – 0.74)  0.53 (0.41 – 0.68)  (2) Cancer  Total W  0.75 (0.63 – 0.88)  Iraq WM  0.33 (0.15 – 0.73)  0.35 (0.19 – 0.66)  Middle East W  0.34 (0.16 – 0.71)  North Africa W  0.41 (0.18 – 0.92)  (3) Cardiovascular  Total M  0.68 (0.55 – 0.84)  F. Yugoslavia M  0.73 (0.58 – 0.93)  Middle East M  0.43 (0.21 – 0.86) | Danish | 1+9 |
|  |  |  | “Overall, immigrant women… and men… also showed significantly decreased all-cause mortality rates compared with native Danes. All ethnic sub-groups also showed significantly lower rates, apart from women from Sub-Saharan Africa, and men from Eastern Europe and Sub-Saharan Africa, whose rate did not differ significantly.” | **Immigrants** | (1) All-cause  Sub-Saharan Africa W  1.12 (0.69 – 1.84)  (2) Cancer  F. Yugoslavia M  1.22 (0.63 – 2.35)  (3) Cardiovascular  Sub-Saharan Africa M  2.06 (0.67 – 6.51) | - | (1) All-cause  East Europe M  0.62 (0.37 – 1.03)  Sub-Saharan Africa M  0.83 (0.49 – 1.41)  (2) Cancer  East Europe WM  0.64 (0.39 – 1.05)  0.43 (0.11 – 1.73)  F. Yugoslavia W  0.46 (0.19 – 1.11)  Iraq WM  0.17 (0.02 – 1.21)  0.99 (0.32 – 3.08)  North Africa M  0.36 (0.12 – 1.12)  Sub-Saharan Africa WM  0.78 (0.25 – 2.41)  1.23 (0.40 – 3.84)  (3) Cardiovascular  East Europe M  0.72 (0.23 – 2.29)  F. Yugoslavia WM  0.69 (0.26 – 1.85)  0.52 (0.17 – 1.64)  Iraq M  0.88 (0.22 – 3.54)  Middle East W  0.53 (0.26 – 1.08)  North Africa WM  0.31 (0.04 – 2.20)  0.88 (0.37 – 2.15) | (1) All-cause  Total WM  0.44 (0.38 – 0.51)  0.43 (0.37 – 0.51)  Asia WM  0.35 (0.26 – 0.48)  0.40 (0.28 – 0.57)  East Europe W  0.47 (0.35 – 0.64)  F. Yugoslavia WM  0.50 (0.32 – 0.79)  0.58 (0.39 – 0.88)  Iraq WM  0.27 (0.12 – 0.61)  0.45 (0.20 – 0.99)  Middle East WM  0.45 (0.34 – 0.59)  0.36 (0.27 – 0.49)  North Africa WM  0.31 (0.16 – 0.60)  0.30 (0.17 – 0.52)  (2) Cancer  Total WM  0.36 (0.26 – 0.48)  0.55 (0.39 – 0.78)  Asia WM  0.23 (0.12 – 0.47)  0.38 (0.17 – 0.85)  Middle East WM  0.31 (0.17 – 0.59)  0.42 (0.22 – 0.82)  (3) Cardiovascular  Total WM  0.39 (0.25 – 0.61)  0.57 (0.38 – 0.84)  Asia WM  0.34 (0.13 – 0.91)  0.25 (0.08 – 0.77)  East Europe W  0.31 (0.11 – 0.83)  Middle East M  0.49 (0.24 – 1.00) | Danish | 1+9 |
| 26. | Cancer mortality | Age | “Mortality rate ratios (MRR) were significantly lower in Turkish immigrants when compared to the majority populations of all four host countries.” | *Reporting:*  *Age-adjusted mortality rate ratios and 95% confidence intervals* | - | - | Turkish-born M  0.85 (0.67-1.08) | Turkish-born W  0.57 (0.41-0.78) | Danish | 1 |
| 27. | (1) All-cause mortality  (2) Other causes of mortality:  Infectious/  parasitic disease  HIV  Other infections  Neoplasm  Mental/ behavioural  Circulatory  Perinatal  Other disease  Accident  Suicide  Other external | - | Low-income countries:  “Our findings indicate that women born in low-income countries are at highest risk of dying during reproductive age in Sweden.”  Middle-income countries:  “The lower mortality risk among women born in middle-income countries may account for the ‘healthy migrant effect’, i.e. people who migrate are healthier than average, and the ‘unhealthy re-migrant’ hypothesis, i.e. immigrants with low mortality risk are continually selected for remaining in the host country.”  High-income countries:  “An increased risk of death in women of reproductive age was also seen in women born in high-income countries.” | *Reporting:*  *Relative risk and 95% confidence intervals*  (1) All-cause  *Low-income*  1.5 (1.3–1.7)  *High-income*  1.2 (1.2–1.3)  (2) Other  *Low-income*  Infectious  15.0 (10.8–20.7)  HIV  39.2 (24.8–61.9)  Other infections  7.1 (4.2–12.0)  Neoplasm  1.3 (1.1–1.6)  Perinatal  6.6 (2.6–16.5)  Other disease  1.4 (1.1–1.9)  Other external  2.1 (1.9–2.3)  *Middle-income*  Infectious  1.5 (1.1–2.1)  HIV  2.7 (1.6–4.7)  Other external  1.5 (1.2–1.8)  *High-income*  HIV  2.7 (1.5–4.9)  Mental  2.4 (1.9–3.0)  Other disease  1.2 (1.1–1.3)  Accidents  1.3 (1.1–1.5)  Suicide  1.6 (1.4–1.8)  Other external  1.9 (1.6–2.3) | (2) Other  *Low-income*  Circulatory  1.4 (1.0–2.0)  Suicide  1.1 (0.8–1.6)  *Middle-income*  Other infections  1.1 (0.7–1.7)  Perinatal  1.1 (0.5–2.6)  *High-income*  Infectious  1.4 (1.0–2.0)  Circulatory  1.2 (1.0–1.3) | (2) Other  *Middle-income*  Neoplasms  1.0 (0.9–1.1)  *High-income*  Other infections  1.0 (0.6–1.7)  Neoplasms  1.0 (0.9–1.1) | (2) Other  *Low-income*  Mental  0.2 (0.0–1.8)  Accidents  0.8 (0.5–1.3)  *Middle-income*  Suicide  0.9 (0.8–1.0)  *High-income*  Perinatal  0.8 (0.2–2.5) | (1) All-cause  *Middle-income*  0.9 (0.8–0.9)  (2) Other  *Middle-income*  Mental  0.3 (0.2–0.6)  Circulatory  0.7 (0.6–0.9)  Other disease  0.7 (0.6–0.8)  Accidents  0.7 (0.6–0.9) | Swedish | 3 |
| 28. | Prostate cancer mortality | Age  Study period  Region | “Overall HRs for PC mortality did not differ between immigrants and Swedes... However, HRs was significantly lower for the low-risk non-European groups.” | *Reporting:*  *Hazard ratio and 95% confidence intervals* | Groups  Other immigrants  1.01 (0.90–1.14) | - | Foreign-born  0.96 (0.91–1.01)  Groups  Low-risk Europeans  0.97 (0.92–1.02)  Low-risk non-Europeans  0.93 (0.68–1.28) | Groups  Very low-risk non-Europeans  0.60 (0.45–0.81) | Swedish | 1+4 |
| 29. | Breast cancer mortality | Age  Study period  Region  Parity  Age at first childbirth | “Among the lowest-risk non-European immigrants… [and] the high-risk immigrants… The HRs were similar and not statistically significant.” | *Reporting:*  *Hazard ratio and 95% confidence intervals* | Foreign-born  1.03 (0.99–1.07)  Groups  High-risk immigrants  1.22 (0.78–1.92)  Low-risk non-Europeans  1.20 (0.92–1.56)  Lowest-risk non-Europeans  1.24 (0.94–1.63)  Other immigrants  1.02 (0.98–1.07) | - | Groups  Low-risk Europeans  0.98 (0.81–1.18) | - | Swedish | 1+5 |
| 30. | Injury mortality:  (1) Suicide  (2) Homicide  (3) Un-intentional injuries | Age  Income | “Our main results show that compared with native Danes, suicide mortality was significantly lower among male refugees and immigrants, whereas their female counter-parts showed no significant differences. However, immigrant women had a significantly higher homicide rate than native Danes did: no other groups showed significant differences. Last, refugees and immigrants of both sexes had lower mortality from unintentional injuries compared with native Danes.” | *Reporting:*  *Rate ratio and 95% confidence interval*  *Note: For region-specific analyses, only significant findings reported*  **Refugees**  (2) Homicide  Sub-Saharan Africa W  8.55 (1.09-67.21) | (2) Homicide  Total WM  1.71 (0.38-7.77)  2.03 (0.87-4.77) | - | (1) Suicide  Total W  0.80 (0.40- 1.61) | (1) Suicide  Total M  0.38 (0.24-0.61)  F. Yugoslavia M  0.54 (0.31-0.94)  Iraq M  0.11 (0.01-0.75)  (3) Unintentional injuries  Total WM  0.44 (0.23-0.83)  0.40 (0.29-0.56)  F. Yugoslavia M  0.39 (0.24-0.61)  Iraq M  0.30 (0.13-0.68) | Danish | 4 |
|  |  |  |  | **Immigrants**  (2) Homicide  Total W  3.09 (1.11-8.60) | - | - | (1) Suicide  Total W  0.87 (0.46-1.65)  (2) Homicide  Total M  0.51 (0.07-3.92) | (1) Suicide  Total M  0.24 (0.10-0.59)  Middle East M  0.22 (0.05-0.87)  (3) Unintentional injuries  Total WM  0.40 (0.21-0.76)  0.22 (0.12-0.42)  Middle East M  0.27 (0.11-0.61) | Danish | 2 |
| 31. | (1) Circulatory disease mortality  (2) Ischaemic heart disease mortality  (3) Cerebro-vascular disease mortality | Age  Sex | Circulatory:  “We observed a consistently high mortality in the South Asia and Eastern Europe-born in Denmark… and Sweden (Eastern Europeans only)… Other COBs experiencing high mortality included the Middle East-born in Denmark... In contrast, we found low mortality in East Asians in… Sweden.”  Ischaemic:  “We observed generally high mortality from IHD among South Asians and Eastern Europeans in Denmark… and Sweden (Eastern Europeans only). Excess deaths were also noted for the Middle East-born in Denmark...”  Cerebrovascular:  “We noted overall high mortality from cerebrovascular disease among  Southeast Asians in Denmark… and Sweden; South Asians in Denmark… Eastern Europeans in Denmark… and Sweden and the  Middle East-born in Denmark.” | *Reporting:*  *Mortality rate ratios and 95% confidence intervals*  (1) Circulatory  South Asia  1.91 (1.58–2.30)  Eastern Europe  1.51 (1.35–1.69)  Middle East  1.49 (1.31–1.69)  (2) Ischaemic  South Asia  2.02 (1.57–2.59)  Eastern Europe  1.39 (1.18–1.63)  Middle East  1.77 (1.51–2.07)  (3) Cerebro-vascular  South Asia  1.92 (1.28–2.89)  Southeast Asia  1.93 (1.25–3.00)  Eastern Europe  1.55 (1.22–1.97) | (3) Cerebro-vascular  Middle East  1.24 (0.91–1.68) | - | (1) Circulatory  Southeast Asia  0.91 (0.68–1.22) | (2) Ischaemic  Southeast Asia  0.42 (0.22–0.80) | Danish | 4 |
|  |  |  |  | (1) Circulatory  Eastern Europe  1.05 (1.03–1.08)  (2) Ischaemic  Eastern Europe  1.08 (1.04–1.12)  (3) Cerebrovascular  Southeast Asia  2.00 (1.42–2.81)  Eastern Europe  1.08 (1.01–1.15) | (2) Ischaemic  South Asia  1.11 (0.83–1.48)  (3) Cerebrovascular  East Asia  1.34 (0.92–1.96)  East Sub-Sahara  1.12 (0.74–1.70) | - | (1) Circulatory  East Asia  0.64 (0.51–0.81)  South Asia  0.99 (0.78–1.26)  Southeast Asia  0.80 (0.63–1.02)  (2) Ischaemic  Southern Latin America  0.32 (0.10–1.01)  (3) Cerebrovascular  South Asia  0.74 (0.39–1.43)  Southern Latin America  0.32 (0.04–2.29) | (1) Circulatory  Southern Latin America  0.37 (0.17–0.83)  Middle East  0.84 (0.79–0.89)  East Sub-Sahara  0.73 (0.59–0.92)  (2) Ischaemic  East Asia  0.46 (0.32–0.68)  Southeast Asia  0.40 (0.25–0.63)  Middle East  0.92 (0.85–0.99)  East Sub-Sahara  0.62 (0.45–0.86)  (3) Cerebrovascular  Middle East  0.74 (0.63–0.86) | Swedish | 7 |
| 32. | Cancer mortality:  (1) All-site  (2) Colon  (3) Lung  (4) Stomach  (5) Prostate  (6) Breast  (7) Cervical | Education  Age (follow-up)  Calendar period at baseline | All-site:  “Compared with  Sweden-born men and women, all-site cancer MRs were lower in their foreign-born counterparts.”  Colon:  “At the country level, a decreased or similar risk of colon cancer mortality was found among men and women born in all studied countries except men born in Latvia, in whom the risk was elevated by 40%, and women born in Denmark, who had a 30% elevated risk.”  Lung:  “The risk remained higher in foreign-born individuals even after multivariable adjustment for education level, age, and calendar period at baseline.”  Stomach:  “Overall foreign-born men and women had similar risks of stomach cancer mortality compared with Sweden-born men and women, respectively after multivariable adjustment for education level, age, and calendar period at baseline.”  Prostate:  “Foreign-born men had an overall 30% lower risk of prostate cancer mortality compared with Sweden-born men after adjustment for education level, age, and calendar period at baseline.”  Breast:  “Compared with Sweden-born women, foreign-born women had an overall 8% lower risk of breast cancer mortality after adjustment for level of education, age, and calendar period at baseline.”  Cervical:  “In contrast to breast cancer, the cervical cancer MR and MRR were both higher in foreign-born women compared to those born in Sweden.” | *Reporting:*  *Mortality rate ratios and 95% confidence intervals for women (W) and men (M)*  *Note: Only continental/ regional results reported here (see paper for country-specific results)*  (1) All-site  Total M  1.05 (1.04–1.07)  (3) Lung  *Foreign-born WM*  1.34 (1.29–1.40)  1.65 (1.60–1.69)  West Asia M  1.35 (1.19–1.54)  *Total Europe WM*  1.37 (1.32–1.43)  1.70 (1.65–1.75)  East Europe WM  1.24 (1.12–1.38)  1.55 (1.44–1.66)  North Europe WM  1.46 (1.40–1.52)  1.78 (1.72–1.83)  South Europe M  1.84 (1.71–1.97)  West Europe WM  1.13 (1.02–1.25)  1.41 (1.31–1.51)  (4) Stomach  *Total Asia W*  1.26 (1.00–1.58)  East Asia W  1.72 (1.05–2.80)  Southeast Asia W  1.85 (1.12–3.06)  East Europe M  1.18 (1.05–1.33)  South Europe M  1.15 (1.01–1.32)  North Europe W  1.06 (1.00–1.13)  *Total Latin America M*  1.53 (1.12–2.08)  South America M  1.67 (1.21–2.31)  (6) Breast  *Total North America*  1.21 (1.05–1.40)  (7) Cervical  *Foreign-born*  1.22 (1.14–1.32)  *Total Europe*  1.24 (1.15–1.34)  North Europe  1.31 (1.20–1.43) | (2) Colon  East Asia W  1.01 (0.61–1.66)  East Europe WM  1.01 (0.89–1.14)  1.02 (0.89–1.16)  (3) Lung  *Total Africa M*  1.07 (0.86–1.33)  North Africa M  1.17 (0.85–1.61)  Southern Africa M  1.34 (0.64–2.81)  West Africa M  1.64 (0.95–2.84)  *Total Asia M*  1.01 (0.91–1.13)  East Asia W  1.25 (0.85–1.84)  Southeast Asia M  1.17 (0.81.1.68)  South Europe W  1.05 (0.91–1.21)  *Total Latin America W*  1.03 (0.79–1.34)  Central America W  1.30 (0.54–3.15)  (4) Stomach  *Foreign-born W*  1.03 (0.98–1.09)  East Asia M  1.06 (0.62–1.81)  West Asia WM  1.29 (0.95–1.75)  1.12 (0.87–1.43)  *Total Europe WM*  1.05 (0.99–1.10)  1.01 (0.97–1.06)  East Europe W  1.12 (0.97–1.30)  North Europe M  1.03 (0.97–1.09)  South Europe W  1.17 (0.96–1.42)  *Total Latin America W*  1.04 (0.70–1.56)  (5) Prostate (M)  Caribbean  1.78 (0.74–4.29)  (6) Breast  East Africa  1.11 (0.77–1.61)  Southern Africa  1.50 (0.67–1.34)  Central America  1.15 (0.54–2.44)  (7) Cervical  East Asia  1.04 (0.46–2.34)  Southeast Asia  1.46 (0.81–2.61)  East Europe  1.18 (0.95–1.47)  South Europe  1.04 (0.79–1.37)  West Europe  1.02 (0.83–1.26)  *Total Latin America*  1.14 (0.69–1.89)  South America  1.01 (0.58–1.76)  *Total North America*  1.01 (0.66–1.55) | (1) All-site  Total W  1.0 (0.98–1.01)  (2) Colon  *Total North America W*  1.00 (0.81–1.23)  (4) Stomach  South America W  1.00 (0.65–1.55)  (5) Prostate  Southern Africa  1.00 (0.41–2.42) | (2) Colon  *Total Africa M*  0.70 (0.45–1.11)  North Africa M  0.59 (0.28–1.24)  East Asia M  0.84 (0.47–1.50)  Southeast Asia WM  0.82 (0.46–1.48)  0.82 (0.46–1.48)  West Europe WM  0.93 (0.83–1.04)  0.92 (0.81–1.05)  *Total Latin America WM*  0.61 (0.46–1.00)  0.69 (0.45–1.07)  South America WM  0.66 (0.44–1.00)  0.70 (0.45–1.10)  *Total North America M*  0.94 (0.74–1.18)  (3) Lung  *Total Africa W*  0.71 (0.54–1.12)  East Africa WM  0.67 (0.36–1.22)  0.75 (0.49–1.15)  East Asia M  0.71 (0.48–1.05)  Southeast Asia W  0.83 (0.54–1.29)  *Total Latin America M*  0.95 (0.77–1.18)  Central America M  0.88 (0.36–2.12)  South America WM  0.92 (0.69–1.22)  0.98 (0.78–1.22)  *Total North America WM*  0.94 (0.76–1.17)  0.99 (0.85–1.16)  (4) Stomach  *Foreign-born M*  0.99 (0.95–1.04)  *Total Africa WM*  0.49 (0.21–1.10)  0.76 (0.48–1.18)  East Africa M  0.57 (0.23–1.38)  North Africa M  0.80 (0.41–1.56)  *Total Asia M*  0.89 (0.72–1.10)  South-Central Asia W  0.90 (0.57–1.42)  Southeast Asia M  0.77 (0.34–1.73)  West Europe W  0.88 (0.77–1.02)  *Total North America W*  0.75 (0.56–1.00)  (5) Prostate  *Total Africa*  0.74 (0.49–1.09)  East Africa  0.72 (0.36–1.46)  *Total Latin America*  0.88 (0.65–1.19)  South America  0.73 (0.53–1.03)  *Total North America*  0.90 (0.77–1-04)  (6) Breast  *Total Africa*  0.97 (0.72–1.30)  North Africa  0.78 (0.42–1.46)  South-Central Asia  0.84 (0.65–1.07)  Southeast Asia  0.72 (0.50–1.03)  East Europe  0.94 (0.83–1.03)  South Europe  0.90 (0.80–1.01)  West Europe  0.99 (0.91–1.07)  (7) Cervical  *Total Africa*  0.53 (0.21–1.29)  South-Central Asia  0.51 (0.25–1.03) | (2) Colon  *Foreign-born WM*  0.91 (0.87–0.95)  0.91 (0.86–0.96)  *Total Africa W*  0.49 (0.25–0.96)  *Total Asia WM*  *0.61 (0.48–0.78)*  0.59 (0.46–0.75)  South-Central Asia WM  0.37 (0.22–0.62)  0.59 (0.39–0.90)  West Asia WM  0.56 (0.40–0.78)  0.56 (0.40–0.78)  *Total Europe WM*  0.91 (0.87–0.96)  0.92 (0.87–0.97)  North Europe WM  0.91 (0.86–0.96)  0.92 (0.87–0.98)  South Europe WM  0.67 (0.55–0.82)  0.76 (0.64–0.90)  (3) Lung  *Total Asia W*  0.77 (0.64–0.92)  South-Central Asia WM  0.46 (0.32–0.67)  0.47 (0.36–0.61)  West Asia W  0.71 (0.55–0.90)  (4) Stomach  South-Central Asia M  0.55 (0.35–0.86)  West Europe M  0.75 (0.66–0.86)  *Total North America M*  0.59 (0.45–0.79)  (5) Prostate  *Foreign-born*  0.72 (0.69–0.74)  North Africa  0.42 (0.19–0.88)  *Total Asia*  0.39 (0.31–0.49)  East Asia  0.52 (0.32–0.84)  South-Central Asia  0.42 (0.29–0.62)  West Asia  0.28 (0.20–0.39)  *Total Europe*  0.72 (0.69–0.75)  East Europe  0.60 (0.54–0.67)  North Europe  0.77 (0.74–0.81)  South Europe  0.39 (0.33–0.46)  West Europe  0.75 (0.68–0.82)  (6) Breast  *Foreign-born*  0.92 (0.89–0.95)  *Total Asia*  0.77 (0.66–0.89)  East Asia  0.58 (0.37–0.90)  West Asia  0.79 (0.65–0.96)  *Total Europe*  0.92 (0.88–0.95)  North Europe  0.90 (0.86–0.94)  *Total Latin America*  0.76 (0.59–0.96)  South America  0.72 (0.56–0.94)  (7) Cervical  *Total Asia*  0.68 (0.47–0.98)  Western Asia  0.37 (0.19–0.72) | Swedish | 1+5+15 |
| 33. | Stroke mortality | - | “A significantly higher risk of death from stroke was found in those born in Finland.” | *Reporting:*  *Odds ratios with 95% confidence intervals*  Finland | 1970-1979  Women  1.23 (0.98-1.54)  Men  1.09 (0.82-1.45)  1990-1999  Women  1.07 (0.95-1.21)  Men  1.03 (0.89-1.20) | - | 1980-1989  Women  0.97 (0.83-1.14)  Men  0.95 (0.77-1.16) | - | Swedish | 1 |
| 34. | Stroke mortality  (*Case fatality 8-28 days*  *Case fatality 29 days to 1 year*) | Education  Income group  Living alone  Sex  Level of consciousness on admission  Atrial fibrillation  Diabetes  Smoking  Type of stroke  Year of stroke | “Country of birth was not significant in either of the two phases.” | *Reporting:*  *Odds ratios with 95% confidence intervals* | 8-28 days  Nordic  1.094 (0.834-1.436)  European  1.180 (0.961-1.449)  >28 days  Nordic  1.055 (0.991-1.221) | - | 8-28 days  Other countries  0.583 (0.270-1.259)  >28 days  European  0.948 (0.784-1.146)  Other countries  0.839 (0.579-1.215) | - | Swedish | 3 |
| 35. | Cancer mortality:  (1) Gynae-cological  (2) Breast  (3) Colorectal  (4) Lung | Age  Disease stage  Comorbidity  Income | “Our hypothesis was not confirmed in that cancer mortality was not significantly higher among migrants than among Danish-born patients in any of our analyses.” | *Reporting:*  *Hazard ratios with 95% confidence intervals*  *Note:*  *Sex-specific results not reported here* | (1) Gynaecological  Foreign-born  1.12 (0.70-1.80)  Middle East  1.45 (0.48-4.42)  Eastern Europe  1.19 (0.68-2.08)  (2) Breast  Middle East  1.14 (0.50-2.63)  (3) Colorectal  Foreign-born  1.09 (0.61-1.92)  Eastern Europe  1.21 (0.67-2.19) | - | (1) Gynaecological  Other countries  0.48 (0.11-2.07)  (2) Breast  Foreign-born  0.76 (0.49-1.17)  Eastern Europe  0.69 (0.39-1.22)  Other countries  0.49 (0.15-1.55)  (4) Lung  Foreign-born  0.78 (0.57-1.07)  Eastern Europe  0.83 (0.58-1.18)  Other countries  0.61 (0.24-1.58) | - | Danish | 1+3 |
| 36. | (1) All-cause mortality  (2) Circulatory disease mortality  (3) Neoplasm mortality  (4) External cause mortality | Age  Education  Income  Occupational class position | “We found higher all-cause mortality among many immigrant categories, although some groups had lower mortality. When studying cause specific mortality, we found the largest differentials in deaths from circulatory disease, whereas disparities in mortality from neoplasms were smaller. SEP, especially income and occupational class, accounted for most of the mortality differentials by country of birth.” | *Reporting:*  *Hazard ratios with 95% confidence intervals*  (1) All-cause  Finland WM  1.05 (1.01-1.09)  1.30 (1.26-1.33)  (2) Circulatory  Finland WM  1.21 (1.13-1.29)  1.37 (1.31-1.43)  (3) Neoplasm  Bosnia M  1.34 (1.18-1.52)  Denmark WM  1.27 (1.12-1.43)  1.19 (1.06-1.33)  (4) External  Finland WM  1.45 (1.01-1.62)  1.45 (1.34-1.56) | (1) All-cause  Denmark W  1.14 (1.05-1.24)  Norway W  1.03 (0.95-1.12)  (2) Circulatory  Denmark W  1.02 (0.84-1.24)  Norway W  1.13 (0.94-1.35)  Thailand M  1.02 (0.38-2.70)  (3) Neoplasm  Finland M  1.11 (1.06-1.17)  Yugoslavia M  1.08 (0.98-1.20)  Germany M  1.04 (0.92-1.17)  Norway M  1.06 (0.92-1.23)  Somalia M  1.27 (0.77-2.11)  (4) External  Poland W  1.13 (0.87-1.47)  Denmark W  1.10 (0.77-1.57)  Norway WM  1.15 (0.83-1.60)  1.12 (0.89-1.41) | (1) All-cause  Norway M  1.00 (0.92-1.08) | (1) All-cause  Germany M  0.94 (0.88-1.01)  Denmark M  0.96 (0.90-1.02)  Somalia M  0.93 (0.72-1.20)  Thailand M  0.80 (0.46-1.42)  (2) Circulatory  Iraq W  0.71 (0.48-1.03)  Yugoslavia W  0.86 (0.73-1.01)  Poland M  0.95 (0.79-1.14)  Denmark M  0.95 (0.85-1.07)  Norway M  0.92 (0.79-1.07)  Somalia WM  0.50 (0.20-1.19)  0.73 (0.41-1.33)  Thailand W  0.98 (0.57-1.69)  (3) Neoplasm  Poland WM  0.93 (0.81-1.06)  0.88 (0.72-1.08)  Norway W  0.99 (0.87-1.13)  Somalia W  0.55 (0.28-1.13)  Thailand WM  0.79 (0.52-1.19)  0.73 (0.18-2.90)  (4) External  Germany WM  0.74 (0.50-1.11)  0.91 (0.71-1.17)  Somalia WM  0.60 (0.15-2.41)  0.64 (0.35-1.20)  Thailand WM  0.57 (0.26-1.29)  0.93 (0.30-2.90) | (1) All-cause  Iraq WM  0.51 (0.42-0.61)  0.49 (0.43-0.57)  Yugoslavia WM  0.74 (0.69-0.80)  0.90 (0.85-0.95)  Poland WM  0.85 (0.78-0.93)  0.86 (0.78-0.95)  Iran WM  0.34 (0.29-0.40)  0.41 (0.37-0.46)  Bosnia WM  0.59 (0.53-0.65)  0.73 (0.67-0.79)  Germany W  0.78 (0.72-0.85)  Turkey WM  0.49 (0.42-0.57)  0.76 (0.55-0.69)  Somalia W  0.60 (0.42-0.86)  Thailand W  0.65 (0.50-0.84)  (2) Circulatory  Iraq M  0.63 (0.50-0.80)  Yugoslavia M  0.88 (0.80-0.97)  Poland W  0.77 (0.62-0.96)  Iran WM  0.24 (0.15-0.38)  0.40 (0.32-0.51)  Bosnia WM  0.60 (0.48-0.75)  0.75 (0.65-0.88)  Germany WM  0.75 (0.62-0.90)  0.87 (0.76-0.99)  Turkey WM  0.56 (0.41-0.78)  0.61 (0.50-0.75)  (3) Neoplasm  Finland W  0.94 (0.89-0.99)  Iraq WM  0.73 (0.55-0.97)  0.72 (0.56-0.92)  Yugoslavia W  0.82 (0.73-0.92)  Iran WM  0.44 (0.34-0.57)  0.47 (0.37-0.59)  Bosnia W  0.84 (0.72-0.98)  Germany W  0.82 (0.72-0.92)  Turkey WM  0.63 (0.50-0.80)  0.76 (0.61-0.93)  (4) External  Iraq WM  0.30 (0.26-0.74)  0.37 (0.26-0.53)  Yugoslavia WM  0.65 (0.47-0.89)  0.62 (0.52-0.75)  Poland M  0.70 (0.51-0.95)  Iran WM  0.31 (0.17-0.56)  0.37 (0.30-0.49)  Bosnia WM  0.38 (0.23-0.63)  0.24 (0.16-0.36)  Denmark M  0.51 (0.42-0.74)  Turkey WM 0.41 (0.21-0.79)  0.31 (0.20-0.46) | Swedish | 12 |
| 37. | All-cause mortality | Education  Stratified by:  Duration of stay at cancer diagnosis  Calendar year at diagnosis  Age group at diagnosis | “After 1990, the overall survival disparity between foreign-born and Sweden-born patients started to increase gradually over time, though the patterns were inconsistent among migrant groups with different durations of residency until 2000 when the increase became more evident and steady, and particularly among men.” | *Reporting:*  *Hazard ratios and 95% confidence intervals, by duration of stay, for 2000-2009, age groups 45-74*  *Note:*  *For results on all calendar years and age groups, please see paper*  5-14 years  Age 45-49 W  1.28 (1.01-1.62)  Age 55-59 M  1.46 (1.17-1.82)  Age 60-64 M  1.60 (1.30-1.98)  Age 65-69 M  1.50 (1.23-1.84)  15-29 years  Age 50-54 M  1.22 (1.04-1.44)  Age 55-59 M  1.36 (1.17-1.58)  Age 60-64 M  1.42 (1.21-1.66)  Age 65-69 M  1.47 (1.24-1.74)  Age 70-74 M  1.25 (1.02-1.52)  30+ years  Age 50-54 M  1.23 (1.04-1.45)  Age 55-59 M  1.30 (1.18-1.44)  Age 60-64 M  1.21 (1.12-1.32)  Age 65-69 M  1.33 (1.24-1.42)  Age 70-74 M  1.16 (1.08-1.24) | 5-14 years  Age 45-49 M  1.14 (0.89-1.47)  Age 50-54 WM  1.02 (0.79-1.32)  1.25 (0.99-1.56)  Age 60-64 W  1.22 (0.95-1.57)  Age 65-69 W  1.15 (0.89-1.48)  Age 70-74 WM  1.23 (0.96-1.58)  1.07 (0.85-1.35)  15-29 years  Age 45-49 WM  1.07 (0.89-1.30)  1.19 (0.97-1.44)  Age 70-74 W  1.03 (0.83-1.29)  30+ years  Age 45-49 WM  1.12 (0.86-1.44)  1.17 (0.87-1.57)  Age 60-64 W  1.04 (0.94-1.15)  Age 70-74 W  1.06 (0.98-1.14) | 30+ years  Age 50-54 W  1.0 (0.86-1.18)  Age 65-69 W  1.0 (0.92-1.09) | 5-14 years  Age 55-59 W  0.88 (0.66-1.16)  15-29 years  Age 50-54 W  0.99 (0.83-1.18)  Age 55-59 W  0.92 (0.76-1.10)  Age 60-64 W  0.81 (0.65-1.01)  Age 65-69 W  0.97 (0.78-1.21)  30+ years  Age 55-59 W  0.97 (0.87-1.09) | - | Swedish | 1 |
| 38. | Suicide | Age  Sex  Education  Family situation  Type of residential area  Labour market marginalization variables  Healthcare variables | “First-generation immigrants born in Nordic countries had a 68% higher crude HR. The association decreased in step-wise adjustment and was not signiﬁcant after adjusting for morbidity. Compared with the natives, the suicide risk in the ﬁrst-immigrant generation born in Europe outside EU-25 and in the rest of the world were signiﬁcantly lower in the crude models, and still remained signiﬁcantly lower when all potential confounders were adjusted for.” | *Reporting:*  *Hazard ratios and 95% confidence intervals* | Nordic  1.13 (0.96-1.32) | - | EU 25/Other Western countries  0.93 (0.75-1.15) | Europe outside EU  0.56 (0.46-0.68)  Rest  0.44 (0.33-0.39) | Swedish | 4 |
| 39. | (1) All-cause mortality  (2) Cause-specific:  Suicide  Homicide  Cardio-vascular disease  Respiratory disease  Infectious disease  Cancer  Unintentional injuries  Other causes | Age  Sex (*for cause-specific*) | All-cause:  “Within-country comparisons showed that immigrants in Denmark had an unfavourable mortality pattern, compared to the local-born population.”  Cause-specific:  “Mortality for suicide, respiratory diseases, cancer and unintentional injuries were generally lower in immigrants compared to local-born, with little cross-country differences.” | *Reporting:*  *Mortality rate ratios and 95% confidence intervals*  (1) All-cause  Turkey WM  1.34 (1.15-1.55)  1.52 (1.38-1.67)  Morocco M  1.31 (1.04-1.65)  (2) Cause-specific  *Homicide*  Morocco  5.00 (1.61-15.58)  *Cardiovascular*  Turkey  1.77 (1.51-2.08)  *Other causes*  Turkey  3.45 (3.08-3.87)  Morocco  2.46 (1.81-3.34) | (2) Cause-specific  *Homicide*  Turkey  1.92 (0.86-4.31)  *Infectious*  Morocco  1.08 (0.27-4.30) | - | (1) All-cause  Morocco W  0.88 (0.54-1.41)  (2) Cause-specific  *Suicide*  Morocco  0.48 (0.16-1.50)  *Cardiovascular*  Morocco  0.74 (0.41-1.34)  *Respiratory*  Turkey  0.77 (0.55-1.07)  Morocco  0.80 (0.36-1.78)  *Infectious*  Turkey  0.77 (0.37-1.61)  *Cancer*  Morocco  0.84 (0.54-1.32)  *Unintentional injuries*  Morocco  0.96 (0.52-1.78) | (2) Cause-specific  *Suicide*  Turkey  0.42 (0.24-0.72)  *Cancer*  Turkey  0.70 (0.57-0.86)  *Unintentional injuries*  Turkey  0.35 (0.23-0.56) | Danish | 2 |
| 40. | Lung cancer mortality | Age  Sex  Family income  Marital status  Education  Region of residence  Mobility  Comorbidities | “The highest odds of mortality were found for individuals who were men, never married, widowed, or divorced, immigrants, those who had a middle-level family income, had the lowest educational attainment, or were affected with comorbidities.” | *Reporting:*  *Odds ratios and 95% confidence intervals*  Foreign-born  1.18 (1.14-1.22) | - | - | - | - | Swedish | 1 |
| 41. | (1) All-cause mortality  (2) NCD mortality:  Cardio-vascular  Cancer  Diabetes  Chronic respiratory  All NCDs  (3) Other | Age (decades)  Calendar time (4-year periods) | “The in-migrant group experienced a mortality advantage in earlier periods compared with those born in Sweden, but this reduced to a nil difference in the final time period.” | *Reporting:*  *Adjusted mortality rate ratios and 95% confidence intervals*  *Note:*  *Foreign-born or “in-migrant” used as reference group, reported values on Swedish-born individuals* | - | - | - | (1) All-cause  1.42 (1.40-1.44)  (2) NCD  Cardiovascular  1.49 (1.45-1.52)  Cancer  1.39 (1.36-1.42)  Diabetes  1.73 (1.58-1.90)  Chronic respiratory  1.87 (1.73-2.02)  All NCD  1.46 (1.44-1.48)  (3) Other causes  1.33 (1.30-1.46) | Foreign-born | 1 |
| 42. | Cutaneous malignant melanoma (CMM) mortality | Gender  Age at diagnosis  Education  Living area  Tumour site  Healthcare region | “Overall, there was no signiﬁcant difference between the entire group of immigrants compared to Swedish-born patients… The CMM-speciﬁc survival was signiﬁcantly reduced among women born in Former Yugoslavia compared to Swedish-born women.” | *Reporting:*  *Hazard ratios and 95% confidence intervals*  *Note:*  *Sex-specific results for Yugoslavia reported in-text* | Southern Europe  1.03 (0.67–1.59)  Former Yugoslavia  1.18 (0.75–1.86) | Other Nordics  1.00 (0.84–1.20) | Foreign-born  0.94 (0.82–1.07)  Western Europe  0.92 (0.63–1.32)  Eastern Europe  0.79 (0.54–1.14)  Non-European  0.69 (0.40–1.18)  Southern Europe (excl. Former Yugoslavia)  0.49 (0.12–1.97) | - | Swedish | 1+7 |
| 43. | All-cause mortality | Age group  Marital status  Parental status  Education | “After adjustments for parental status, marital status, and educational level, hereafter referred to as sociodemographic factors, the survival advantage of immigrants as a group compared to hosts became more pronounced (OR 0.81, CI 0.79–0.82), but, relative to hosts, the effect appeared similar for male (OR 0.81, CI 0.79–0.83) and female (OR 0.82, CI 0.80–0.84) immigrants.” | *Reporting:*  *Odds ratios and 95% confidence intervals* | - | Nordic W  1.00 (0.96 – 1.04) | North America/  Oceania W  0.96 (0.89 – 1.04) | Foreign-born WM  0.82 (0.80 – 0.84)  0.81 (0.79 – 0.83)  Nordic M  0.95 (0.92 – 0.98)  West Europe WM  0.93 (0.89 – 0.98)  0.75 (0.72 – 0.79)  East Europe WM  0.70 (0.65 – 0.74)  0.79 (0.75 – 0.83)  Middle East WM  0.49 (0.44 – 0.55)  0.70 (0.65 – 0.75)  South Asia WM  0.63 (0.57 – 0.69)  0.77 (0.72 – 0.82)  Asia WM  0.54 (0.50 – 0.59)  0.61 (0.56 – 0.67)  Africa WM  0.69 (0.60 – 0.78)  0.76 (0.70 – 0.84)  North America/ Oceania M  0.85 (0.79 – 0.92)  Other WM  0.62 (0.54 – 0.71)  0.68 (0.61 – 0.76) | Norwegian | 1+16 |
